# Developmental Features of Lexical Richness in English Writings by Chinese Beginner Learners

**DOI:** 10.3389/fpsyg.2021.665988

**Published:** 2021-06-02

**Authors:** Huiping Zhang, Ming Chen, Xuelan Li

**Affiliations:** School of Foreign Languages, Northeast Normal University, Changchun, China

**Keywords:** developmental features, lexical richness, L2 writings, Chinese beginner learners, language exposure hypothesis

## Abstract

This paper reports a cross-sectional study that investigates the developmental features in second language writings by Chinese beginner learners of English by using four lexical richness measures—lexical sophistication, lexical variation, lexical density, and lexical errors—from the perspective of the language exposure hypothesis. Specifically, the study compares English compositions written by Chinese students of grade 7, grade 8, and grade 9 in terms of lexical sophistication, lexical variation, lexical density, and lexical errors. The English compositions were sampled from the Writing Corpus of Chinese Beginner Learners of English, and the sample size of the three grades remained almost the same. The analysis revealed that lexical richness in the writing samples of beginner learners is comparatively low, with beginner learners transferring lexical features of the oral register to their second language writings; furthermore, all four measures yielded significant, albeit non-linear and unevenly paced, developments across grade levels. Based on the findings, several suggestions for vocabulary teaching are provided.

## Introduction

Lexical richness as a multidimensional construct in second language (L2) learning (Schmitt, 2014) has been recognized as an integral component for the construction of written products (Engber, 1995; González, 2017). Well-written texts are involved in writer’s purposeful selection and use of lexis. A sophisticated, diverse, and accurate lexical contribution to texts enhances writing quality and mirrors the learner’s writing proficiency (e.g., Laufer and Nation, 1995; Read, 2000; Qin and Wen, 2007). Therefore, lexical richness is a crucial aspect in L2 writing research. Previous studies of lexical richness in L2 writings can be divided into three categories. Research in the first category tries to identify the relationship between lexical richness measures and L2 writing quality. These studies involve a series of correlation and regression analyses and yield mixed results regarding different measures (e.g., Engber, 1995; Qin and Wen, 2007; Wang and Zhou, 2012; Zhu, 2013; Lee et al., 2021; Schnur, 2021). The studies in the second category explore the level of L2 learners’ lexical richness compared with that of native English speakers (e.g., Fairclough and Belpoliti, 2016; Eckstein and Ferris, 2018; Deng, 2020; Lei and Yang, 2020).

Given the role of lexical richness in determining writing quality, research in the third category selects validated indices and analyzes lexical richness in L2 writing from a developmental perspective. The bulk of research along this line has dealt with one or two measures, together with other linguistic features (e.g., Bulté and Housen, 2014; Hsieh, 2016; Tracy-Ventura, 2017; Zhang and Daller, 2020). For instance, lexical variation and syntactic complexity have been measured in academic writings by adult English as a second language learner (Bulté and Housen, 2014) and in narrative texts by primary school students (Hsieh, 2016). Lexical sophistication has been examined solely in L2 learners’ Spanish oral and written data before and after their study abroad (Tracy-Ventura, 2017). In contrast, little evidence is available in a holistic analysis of each lexical richness measure. Of the few studies that have examined lexical richness holistically, most have focused predominantly on intermediate and advanced L2 learners and produced rather inconsistent findings. For instance, Bao (2008) compared lexical differences in L2 English compositions among university students at three proficiency levels. The results demonstrated that lexical richness measures increase to different degrees with proficiency level: lexical sophistication increases linearly, while other measures, including lexical variation, lexical density, and lexical originality, develop non-linearly. Wang and Zhou (2012) reported a longitudinal study of lexical growth in university non-English majors. The results revealed that lexical sophistication, lexical variation, and lexical density increase steadily as a function of grade level. Learners commit fewer lexical errors as they become more proficient. However, Zheng (2016) found that lexical density flattened in English compositions by university students during a 1-year reading course, whereas lexical sophistication and lexical variation increased over time.

As is evident from prior studies, research participants in the literature are limited to learners at higher proficiency levels, university students in particular (e.g., Bao, 2008; Zhu and Wang, 2013; Zheng, 2015, 2018; Lei and Yang, 2020). Much remains to be known about how lexical richness develops over time in L2 writings by beginner learners. Considering that age and language proficiency are crucial variables in L2 learning (Ellis, 2013), beginner learners might present developmental features of lexical richness different from intermediate and advanced learners. Moreover, compared with learners at higher proficiency levels, beginner learners are more likely to struggle with sophisticated, varied, and accurate control of vocabulary in L2 writings (Fairclough and Belpoliti, 2016).

For these reasons, it is meaningful to examine the developmental features of lexical richness in L2 writings by beginner learners. Specifically, the present study investigates the developmental features of the four dimensions of lexical richness (namely, Lexical Sophistication, Lexical Variation, Lexical Density, and Lexical Errors) in English compositions by Chinese beginner learners, by performing cross-sectional comparisons between three grade levels of writing. By comparing lexical differences across grade levels, this study can yield insights into the way lexical richness develops in learners’ L2 writing. In addition, it identifies the deficiency in lexical use of this learner population and uncovers their learning needs, which can, in turn, suggest implications for vocabulary teaching in secondary schools. Specifically, it addresses four research questions:

(1) What are the developmental features of lexical sophistication in English compositions by Chinese beginner learners?
(2) What are the developmental features of lexical variation in English compositions by Chinese beginner learners?
(3) What are the developmental features of lexical density in English compositions by Chinese beginner learners?
(4) What are the developmental features of lexical errors in English compositions by Chinese beginner learners?

## Theoretical Framework

Many factors have been argued to be influential in L2 acquisition, including a learner’s age, motivation, personality and aptitude (Ellis, 2013). According to the usage-based approach, one of the most important factors of L2 acquisition is language input or language experience. In line with this concept, the language exposure hypothesis generalized by Ortega (2014) indicates that language exposure is a prerequisite for L2 acquisition and plays a facilitative role in language learning.

The language exposure hypothesis includes two dimensions (Ortega, 2014): linguistic exposure and social exposure. Specifically, in the first dimension, it is assumed that the whole language system consists of several levels: the lexical level, the syntactic level, the discourse level, and the pragmatic level. Language learning at these levels is influenced by the amount of linguistic exposure. In the second dimension, language learning may also relate to social norms and practices by communicating with native speakers and interacting with the native language’s society.

**FIGURE 1:**
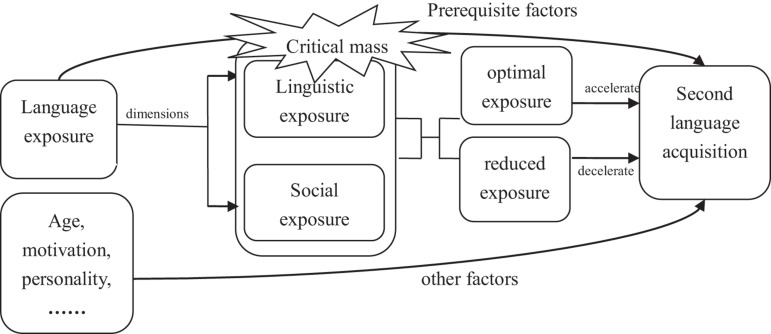
Language exposure hypothesis.

As can be seen in Figure 1, optimal exposure accelerates L2 acquisition, while reduced exposure decelerates L2 acquisition and causes linguistic and social duress. It should be emphasized that linguistic rules can be successfully acquired only if language exposure reaches “critical mass.” Critical mass is often related to the clarity and obscurity of linguistic structures: a transparent linguistic structure requires relatively less critical mass than an obscure one. The most effective way of reaching “critical mass” is raising language exposure frequency in multiple contexts through the increase in linguistic and social exposure.

As the amount of linguistic exposure plays a significant role in lexical learning (Schmitt, 2000), this study adopts the language exposure hypothesis as a theoretical framework to explain the developmental features of beginner learners’ lexical richness.

Chinese learners learn English mainly from classroom teaching and have a very limited exposure to English society. Therefore, this study mainly focuses on the effect of linguistic exposure on L2 acquisition.

## Lexical Richness Measures

**FIGURE 2:**
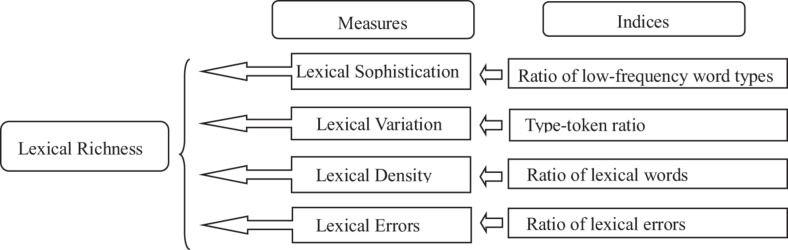
Multidimensional model of lexical richness.

In the literature, the conceptualization of lexical richness has been subject to considerable discussion (see Laufer and Nation, 1995; Jarvis, 2013). Some researchers define lexical richness as equivalent to lexical diversity and lexical complexity (Daller et al., 2003). However, Engber (1995) considers that lexical richness includes lexical variation with error, lexical variation without error, percentage of lexical error, and lexical density. These two definitions are not so superordinate as to reflect the whole situation of vocabulary use in L2 writing. Laufer and Nation (1995) define four dimensions of lexical richness, namely, lexical variation, lexical density, lexical sophistication, and lexical originality. In light of the lexical features of L2 writing, Read (2000) points out that lexical originality could not evaluate the development of lexical performance. He takes error into consideration and modifies Laufer and Nation’s (1995) categorization by defining lexical richness as a superordinate term for the effective use of vocabulary in good writing. It is composed of lexical sophistication, lexical variation, lexical density, and lexical errors, each of which can be independently evaluated (see Figure 2). Therefore, the present study follows this multidimensional model and traces developmental features of the four measures in English compositions by Chinese beginner learners. The following subsections give a brief overview of each measure and present the indices with which the measures are calculated.

### Lexical Sophistication

Lexical sophistication measures “the proportion of relatively unusual or advanced words in the learner’s text” (Read, 2000, p. 203). It is indicative of L2 writing quality (Read, 2000; Zhu, 2013). A well-written text is supposed to involve a number of advanced words that fit in with particular genres because the selection of advanced words, rather than general terms, enables learners to articulate their ideas in a more precise and unambiguous manner (Read, 2000; Qin and Wen, 2007). Furthermore, lexical sophistication also distinguishes the degree of formality of L2 writing (Qin and Wen, 2007; Zhu, 2013). The more common and general the words are, the more informal and spoken-like the L2 writing will be.

In the extant literature, unusual or advanced words have been generally conceptualized in terms of their frequency (e.g., Laufer and Nation, 1995; González, 2017; Tracy-Ventura, 2017). That is, high-frequency words are taken as basic terms, whereas low-frequency words are considered to be advanced. One widely accepted measure based on frequency is Lexical Frequency Profile (LFP) (Laufer and Nation, 1995). LFP comprises four frequency bands: the first 1,000 most frequent words, the second 1,000 most frequent words, the Academic Word List (AWL) (Coxhead, 2000), which refers to the list of academic vocabulary, and not-in-the-lists words. Laufer (1995) defines words in the AWL and not in the lists that are correctly used by learners as low-frequency words. Accordingly, the ratio of low-frequency word families in the text is an indicator of lexical sophistication. However, Liang et al. (2010) points out that the lemmas of word types are a more reliable counting unit than word families when lexical sophistication is computed. Therefore, the present study took the lemma of word types as its unit.

It is also noteworthy that, as words in LFP go far beyond participants’ lexical proficiency, the initial frequency bands (Laufer and Nation, 1995) are unsuitable for measuring lexical sophistication in beginner learners’ writing. Thus, the present study develops a new profile specifically designed for Chinese beginner learners. The new profile is described in more detail in Section “Data and Methods.” Following Laufer’s (1995) definition, we conceptualized words correctly used in word list 3 (see next section) and not in the lists as low-frequency words. The Range program (Heatley et al., 2002) was used to analyze the coverage of writing samples by low-frequency words and compute lexical sophistication thereafter.

### Lexical Variation

Lexical variation, also known as lexical diversity (see Zheng, 2016; Treffers-Daller et al., 2018), refers to “the range of different words used in a text” (McCarthy and Jarvis, 2010, p. 387). Read (2000) defines it as a variety of different words rather than a limited number of words used repetitively. It is a good diagnostic of learners’ productive word repertoires (e.g., Read, 2000) and L2 writing quality (e.g., González, 2017). Learners with diverse lexical repertoire are inclined to vary their choice of words in L2 writing. Texts of high quality are characterized by a wide range of words rather than a narrow range of words used repetitively, as repetition of particular lexical items would make the texts monotonous (Fairclough and Belpoliti, 2016).

There are different indices for measuring lexical variation; type-token ratio (TTR) is widely used. Types refer to the different lexical words used in the text, while tokens are all the lexical words used in the text, including repetitions of words (Liang et al., 2010). TTR is a validated measure to assess lexical variation, when text length remains constant (Treffers-Daller et al., 2018). In addition, TTR is much easier to calculate than other indices, such as the Uber index [see Jarvis, 2002, p. 59; Uber index: *U* = (logTokens)^2^/(logTokens - logTypes)]. Therefore, this study kept sample sizes of grades 7–9 almost the same and adopted TTR to measure the lexical variation of beginner learners’ writings.

### Lexical Density

Lexical density, defined as “the proportion of lexical words in a text” (Read, 2000, p. 203), reflects the information load of a text (Biber et al., 2012). Since lexical words, as opposed to function words, encode the bulk of the propositional content of the message, a large proportion of lexical words in a text indicates its high information load. Halliday (2002) points out that lexical density is a useful measure to distinguish levels of texts along the oral–written continuum. As written language is “reflective” rather than “active” in nature (Halliday, 2002, p. 329), it packs denser information than its spoken counterpart. Therefore, a text with a higher lexical density is characteristically more written-like, and lexical density will increase if the text moves along the continuum from spoken to written.

### Lexical Errors

The occurrence of errors is a common feature of L2 writing (Read, 2000). Lexical errors in a text could obscure the writer’s original meaning and hinder the reader’s interpretation of the text. Therefore, the percentage of lexical errors is negatively correlated with L2 writing quality (Pilar and Llach, 2007; Wang and Zhou, 2012), especially in the case of less advanced learners (Engber, 1995).

In the literature, a generally accepted definition of lexical errors is lacking (Read, 2000). Researchers have categorized lexical errors in widely varying manners depending on their perceptions (e.g., Engber, 1995; Pilar and Llach, 2007; Wan, 2010). However, in spite of the various taxonomies, L2 learners were unanimously reported to have problems with the formal aspects of the target language (see Schmitt, 2014). Misspellings, among all the categories analyzed, turned out to be the most frequent errors in the beginner learner’s production (e.g., Pilar and Llach, 2007; Wang and Zhou, 2012). Misspellings, even minor misspellings, have the same weight as any other kinds of lexical errors, such as word choices, for they all might make a sentence difficult to interpret (Read, 2000). The frequent occurrence of misspellings shows evidence of the learner’s unfamiliarity with the orthographic features of the target language (Pilar and Llach, 2007; Schmitt, 2014). Additionally, it affects the intelligibility of the message and interferes with the information transmission of written texts. Thus, the present study focuses on misspellings in the beginner learner’s writing and investigates ways that the proportion of misspellings varies over time.

## Materials and Methods

### Corpus Description

The writing samples used in this study were drawn from the Writing Corpus of Chinese Beginner Learners of English. This corpus was compiled by the School of Foreign Languages at Northeast Normal University in the spring semester of 2014. The corpus consists of compositions written by randomly selected secondary school students from mainland China. The compositions in the corpus are descriptive and argumentative. The topics are the same, and they are familiar to the participants: my birthday, my school, my favorite food, a funny story in my life, how to spend money, on smoking, on exercising, and on Watching TV. Participants were given these topics simultaneously and chose one of these topics to complete a writing ranging from 50 to 100 words during a 20-min period without the aid of any reference tools. Altogether, the corpus contains 2,891 pieces of written texts. The total number of tokens in the corpus is 321,759.

For the purpose of this study, compositions by junior high school students (grades 7–9) were sampled from the corpus. The sample sizes of the three grade levels remained almost the same. The samples of grades 7–9 contained 16,708, 16,733, and 16,726 tokens, respectively.

### Participants

The writing samples used in this study were written by Chinese students at junior high schools. Most of these students grew up in the northeast of China, and their L1 was Mandarin Chinese. English is a major subject in junior high schools’ curricula with six or seven periods of instruction each week. The instruction of English writing is an important part of English teaching, with one or two periods every 2 weeks.^1^ Scores in English writing occupy a high percentage of the assessment of students’ English ability (see Ministry of Education of the People’s Republic of China, 2017). Before grade 7, students have received formal English classroom instruction for an average of 3 years with three class periods each week. Most of them merely learned alphabets and could only make daily conversations (Jiang et al., 2019). Their formal learning of English writing begins from grade 7, which is their first year in junior high school. Therefore, junior high school students are regarded as the beginner learners of English writing in this study. It should be emphasized that all the participants had been informed of our academic purpose of collecting their writings and agreed to participate in the corpus construction project.

### Development of Base Lists for Secondary Students

Developing appropriate base lists is of prime importance to measure lexical sophistication (Liang et al., 2010). As noted earlier, since the accompanying frequency bands (Laufer and Nation, 1995) in the Range program exceeded the participants’ lexical proficiency, advanced words defined by reference to the AWL rarely occurred in the samples across the three grade levels. As a result, the measure based on the original base lists is ineffective at capturing the lexical sophistication development of beginner learners’ writing. For this reason, we established three new base lists tailored to assess the lexical richness of junior high school students.

The new base lists contain vocabulary in English textbooks published by People’s Education Press. The series of textbooks are chosen for two reasons. First, for learners who acquire L2 mainly in the classroom setting, words in their L2 lexicon are mostly traceable to textbook vocabularies (Ellis, 2013). Second, the textbooks were compiled in accordance with *English Curriculum Standards for Compulsory Education* (Ministry of Education of the People’s Republic of China, 2017) and were approved by the National Basic Education Teaching Materials Working Committee. Thus, the vocabulary in the textbooks is scientifically organized in a sequence consistent with learners’ natural order of acquiring lexis, i.e., from basic to advanced or from frequent to infrequent. In the present study, we followed the sequence of vocabulary in textbooks and classified the vocabulary into three frequency bands. Specifically, word lists in textbooks for grades 7–9 corresponded to base list 1, base list 2, and base list 3.

Base lists in the present study were compiled with the lemma of word types as the counting unit. That is, inflected forms, excluding derived forms, were grouped into one lemma. For example, the lemma ADVERTISE has the following inflected members: ADVERTISES, ADVERTISED, and ADVERTISING. However, its derived forms ADVERTISEMENT, ADVERTISEMENTS, ADVERTISER, and ADVERTISERS are subsumed under the lemmas ADVERTISEMENT and ADVERTISER separately.

Lemma-based word lists were produced by means of Lemmatizer, available online at https://www.lextutor.ca/familizer/. After the three word lists were output, they were renamed BASEWRD1, BASEWRD2, and BASEWRD3 and substituted for the original frequency bands. The new base lists consist of 1537, 1527, and 1050 lemmas, respectively.

### Data Analysis

As the size of the samples is considerable, the intragroup heterogeneity might be negligible. Therefore, this study follows the tradition of cross-sectional studies (e.g., Jiang et al., 2019) and uses the writing samples from the different grade levels as cross-sectional data to investigate how and where the written texts are in common or differ and to what extent.

#### Identifying the Developmental Features of Lexical Sophistication

This study took the lemma of word types as its unit and measured lexical sophistication as below:

$$\text{Lexical}\,\mathrm{sophistication}=\frac{\text{Number}\,\text{of}\,\text{the}\,\mathrm{low}-\mathrm{frequency}\,\text{word}}{\text{Total}\,\text{number}\,\text{of}\,\text{the}\,\text{word}\,\text{types}}\times 100\%$$

First, the new base lists for secondary high school students were extracted. Once the writing samples of beginner learners were submitted to the Range program, the software automatically compared the samples against three base lists and generated the Beginner Learners’ Writing Vocabulary List. The vocabulary list comprised four sublists, namely, word list 1, word list 2, word list 3, and not in the lists. The first three lists enumerate words in the samples covered by each base list, while the last one contains words not counted by the base lists.

Second, according to the formula of lexical sophistication, the number of word types per word list and the number of word types in each writing sample were calculated for each grade level. Third, the number of low-frequency word types and their percentages in the number of word types in each writing sample were calculated for each grade level. The lexical sophistication of each grade level was calculated thereafter. Last, log-likelihood (LL) tests^2^ were performed to measure whether differences in lexical sophistication were statistically significant across grade levels.

#### Identifying the Developmental Features of Lexical Variation

This study adopted TTR to measure lexical variation through AntConc 3.5.7 (Anthony, 2004) as below:

T⁢T⁢R=(t⁢y⁢p⁢e/t⁢o⁢k⁢e⁢n)×100%

First, according to the formula of lexical variation, TTR was computed for each grade level. Second, comparisons of TTR were made among samples of the three grades through LL tests to capture the overall developmental trend of lexical variation. Third, the number of types per word list was compared between adjacent grade levels in order to see the detailed developmental features of lexical variation.

#### Identifying the Developmental Features of Lexical Density

In this study, lexical density was calculated by dividing the number of lexical word tokens by the total number of tokens in the text (as shown below). Specifically, lexical words encompassed nouns, verbs, adjectives, and adverbs, among which verbs included *be*, *do*, and *have* as lexical verbs (see Biber et al., 2012).

The calculation of lexical density involved the following steps. First, writing samples from the three grade levels were part of speech (POS) tagged with Treetagger and were proofread with PowerGrep 4 (Liang et al., 2010). Then, AntConc 3.5.7 was used to concord each class of lexical words, count the number of tokens of each class, and compute the total number of lexical word tokens. Next, lexical density was calculated based on the index below:

$$\text{Lexical}\,\mathrm{density}=\frac{\mathrm{Total}\,\mathrm{number}\,\text{of}\,\text{lexical}\,\mathrm{word}\,\mathrm{tokens}}{\text{Total}\,\text{number}\,\text{of}\,\text{tokens}}\times 100\%$$

Specifically, according to the formula of lexical density, the ratio of lexical words was first calculated for each grade. Then, LL was computed to test whether differences in the ratio were statistically significant between adjacent grade levels. Next, distribution of lexical word classes was measured for each grade, and comparisons were made in order to examine the distributional differences of lexical words across grade levels.

#### Identifying the Developmental Features of Lexical Errors

This study focuses on misspellings in the beginner learners’ writings. First, the Range program was used to generate the not in the lists, from which misspellings were manually extracted. Second, all the identified misspellings were classified according to the base lists in which the intended words occurred. Third, the percentage of misspellings was calculated and compared for each base list across grade levels. Fourth, the differences between the three grade levels were identified in terms of the total frequency of misspellings through LL texts. Fifth, the way that the percentage of misspellings per base list varied with grade level was traced.

## Results

Based on cross-sectional comparisons between writing samples from grades 7–9, the following subsections present the developmental features of lexical sophistication, lexical variation, lexical density, and lexical errors separately. Examples from the corpus are provided to exemplify the descriptions where necessary.

### Lexical Sophistication

The first research question investigated the developmental features of lexical sophistication in English writings by Chinese beginner learners. To address this question, the overall level of lexical sophistication in the writing samples of three grades was examined first. Then, the developmental trend of lexical sophistication was investigated across the three grade levels. These results are displayed in Tables 1, 2.

**TABLE 1:** Number and percentage of word types per word list and per grade level.

Word lists		Grade 7 Types/%	Grade 8 Types/%	Grade 9 Types/%
High-frequency words	Word list 1	664/48.68	753/50.67	702/40.96
	Word list 2	275/20.16	323/21.74	392/22.87
	Total	939/68.84	1,076/72.41	1,094/63.83
Low-frequency words	Word list 3	86/6.30	110/7.40	141/8.23
	Not in the lists	Correct 202 (total 339)/14.81	Correct 221 (total 300)/14.87	Correct 338 (total 479)/19.72
	Total	Correct 288 (total 425)/21.11	Correct 331 (total 410)/22.27	Correct 479 (total 620)/27.95
Total number of word types		1364	1486	1714

**TABLE 2:** Log-likelihood (LL) tests for lexical sophistication across grade levels.

Grade levels	Word list 3		Not in the lists		Total (lexical sophistication)	
			
	LL	Sig. (*p*)	LL	Sig. (*p*)	LL	Sig. (*p*)
7–8	−1.25	0.264	0.00	0.965	−0.44	0.507
8–9	−0.69	0.406	−10.81	0.001	−10.19	0.001
7–9	−3.85	0.050	−10.59	0.001	−14.42	0.000

As is shown in Table 1, high-frequency word types account for 68.84, 72.41, and 63.83% of the total number of types in the samples of grades 7–9, whereas low-frequency word types cover 21.11, 22.27, and 27.95%. A comparison of the two sets of data reveals that participants are prone to use high-frequency vocabulary in L2 writing, and their productive control of low-frequency words is still at the beginning level.

In addition, as the low-frequency word ratio denotes the degree of formality of L2 writing (Qin and Wen, 2007; Zhu, 2013), the low ratios of infrequent words in this study indicate that compositions by beginner learners are generally informal in style and spoken-like in register. This result is further supported by qualitative analysis of the samples. As shown in Example (1) below, elements that are predominant in spoken language abound in texts by beginner learners, including undue repetition of modal verbs, recurrent second-person pronouns, imperatives, and exclamations (Biber et al., 2012; Zhu, 2013; Fairclough and Belpoliti, 2016). It seems that formality of writing is one of the biggest challenges for L2 learners.

(1) *You can* exercise by doing like those: *You should* do morning exercise after breakfast. Because, it makes *your* work in the morning relaxing. In the afternoon, after *you* lunch. *You can* go for a walk on the playing, It not only keeps *your* health but also make *you* enjoy some beautiful flowers or grass. *Isn’t that great!* Finally, *do some exercise in the evening.* After dinner, *you can* do some sports, like playing basketball, playing tennis and so on. (080610.txt).

With regard to the developmental trend of lexical sophistication, there was a positive growth in the low-frequency word ratio in the samples, with a percentage of 21.11% for grade 7, 22.27% for grade 8, and 27.95% for grade 9 (see Table 1). The gradual increase in lexical sophistication mirrors previous results (Laufer and Nation, 1995; Fairclough and Belpoliti, 2016; González, 2017; Lee et al., 2021; Schnur, 2021), although previous studies mainly investigated intermediate and advanced learners.

This result suggests that participants increase their use of advanced vocabulary in English writing and that they are gradually able to formulate their ideas in a more precise and sophisticated fashion. See one passage from the lower grade in Example (2) below.

(2) In the world. There are lots of *people* are <2> smoking. But <!> smoke is bad for our <2> health. And <2> health is very important. If you are <!> unhealthy. You cannot *do many thing*. You can’t *play* <2> *something and eat* <2> *something*. If your friend or classmate is <2> smoking. Of <2> course you don’t think he is so *great*. And you think he is <!> unfriendly. So don’t <2> smoke, It’s *good* for you and your family.^3^ (070948.txt).

This text from grade 7, compared with upper grades, contains far more basic vocabulary, such as *people*, *thing*, *do*, *many*, *good*, *great*, etc. These general terms perform similar functions to grammatical words, and they carry little specific information of their own (Halliday, 1989). As a result, the text is rife with these general terms and transmits vague and imprecise information. Nonetheless, in the process of English learning, sophisticated vocabulary in beginner learners’ writing increases in number. With the more advanced words, the information in L2 writing was felt to be more precise and unambiguous, and the English compositions tended toward formal style and the written register over time. This result aligns with the findings from previous studies that lexical sophistication develops with incremental language experience (Bulté and Housen, 2014; Zheng, 2016; Tracy-Ventura, 2017; Lee et al., 2021; Schnur, 2021).

However, the upward trend in lexical sophistication was nonlinear (see Table 2). LL demonstrates that the increase is non-significant from grades 7 to 8 (LL = −0.44) but significant from grades 8 to 9 (LL = −10.19) and from grades 7 to 9 (LL = −14.42). In other words, beginner learners in grade 9 show a rate advantage over those in grades 7 and 8 in their use of infrequent words. A close scrutinization of the two low-frequency word lists reveals that the increases in word list 3 are non-significant between adjacent grade levels (grades 7 and 8: LL = −1.25; grades 8 and 9: LL = −0.69) but significant from grades 7 to 9 (LL = -3.85). In comparison, the increases in not in the lists are non-significant from grades 7 to 8 (LL = 0.00) but significant from grades 8 to 9 (LL = −10.81) and from grades 7 to 9 (LL = −10.59). Taken together, these results indicate that the rate advantage for grade 9 primarily stems from the proportional increase in word types in not in the lists.

To conclude, there was a preponderance of basic over advanced vocabulary in L2 writing by beginner learners. Therefore, their English compositions were generally informal in style and spoken-like in register. However, during 3 years of English learning, beginner learners showed progress in lexical sophistication by increasing their use of advanced vocabulary. Specifically, learners in grade 9 produced a significantly higher proportion of low-frequency words than those in grades 7 and 8.

### Lexical Variation

The second research question examined the developmental features of lexical variation in English writings by Chinese beginner learners. To answer this question, the overall level of lexical variation in the writing samples of the three grades was examined first. Then, the developmental trend of lexical variation was explored across the three grade levels.

Table 3 presents TTR in the samples of the three grades, together with LL between adjacent grade levels. As can be seen from the table, TTR is rather low for all three grades, at 8.16, 8.88, and 10.25%, respectively. These low values indicate that the writing samples are not lexically diverse. Beginner learners produce monotonous English compositions with repetitive use of particular lexical items. This result is consistent with that of Fairclough and Belpoliti (2016) and Schnur (2021), who also found a low level of lexical variation in L2 writings by beginner learners of Spanish.

**TABLE 3:** Type-token ratio (TTR) per grade level.

Grade level	LL	Types	Tokens	TTR
7	Grades 7 and 8: LL = −5.04 Sig. (*p*) = 0.02472	1364	16,708	8.16%
8		1486	16,733	8.88%
	Grades 8 and 9: LL = −15.35 Sig. (*p*) = 0.00005			
9		1714	16,726	10.25%

Regarding the developmental trend of lexical variation, Table 3 shows that TTR increases with grade level (8.16 < 8.88 < 10.25%). LL reveals that the increases are all statistically significant between adjacent grade levels (grades 7 and 8: LL = −5.04; grades 8 and 9: LL = −15.35). This result suggests that beginner learners from grades 7 to 9 strategically vary their lexical choices when constructing English compositions.

In addition, the number of word types per word list was also compared between adjacent grade levels. The results are reported in Table 4. As can be observed, with regards to grades 7 and 8, the number of word types in word list 1 significantly increased from 664 to 753 (LL = −5.46). That is, the TTR of word list 1 significantly increased from grades 7 to 8. In comparison, the number of word types in the other three lists did not vary in any statistically significant way. Specifically, the number of word types rose from 275 to 323 for word list 2 (LL = −3.79), from 86 to 110 for word list 3 (LL = −2.91), and from 202 to 221 for not in the lists (LL = −0.83). With regards to grades 8 and 9, the number of word types in word list 1 declined slightly and non-significantly from 753 to 702 (LL = 1.77). Conversely, the other three lists showed significant increases over time. Specifically, the number of word types increased from 323 to 392 for word list 2 (LL = −6.70), from 110 to 141 for word list 3 (LL = −3.85), and from 221 to 338 for not in the lists (LL = −24.72). Taken together, these results indicate that in spite of the overall upward trend in lexical variation, participants in the three grades have striking differences in lexical choice; that is, learners in lower grade levels (grades 7 and 8) tend to vary basic vocabulary in base list 1 when composing English writing, whereas learners in grade 9 prefer to diversify sophisticated vocabulary beyond base list 1. As a result, the compositions become lexically sophisticated as well as varied over time, which is interestingly in line with the significant increase in lexical sophistication.

**TABLE 4:** Number of word types^4^ per word list and per grade level.

Grade level	Word list 1	Word list 2	Word list 3	Not in the lists (correct vocabulary)
7	664^4^	275	86	202
LL	LL = −5.46 Sig. (*p*) = 0.01944	LL = −3.79 Sig. (*p*) = 0.05169	LL = −2.91 Sig. (*p*) = 0.08801	LL = −0.83 Sig. (*p*) = 0.356
8	753	323	110	221
LL	LL = 1.77 Sig. (*p*) = 0.18379	LL = −6.70 Sig. (*p*) = 0.00965	LL = −3.85 Sig. (*p*) = 0.04970	LL = −24.72 Sig. (*p*) = 0.00000
9	702	392	141	338

To summarize, beginner learners showed a lack of varied lexical use and composed lexically monotonous English compositions. Nevertheless, over the course of L2 development, lexical variation increased significantly, and the compositions became lexically more diverse. In addition, learners in the three grades presented distinctive developmental features when structuring English writing: those in lower grades employed diverse basic vocabulary in base list 1, whereas those in grade 9 used varied sophisticated vocabulary beyond base list 1.

### Lexical Density

The third research question concerned the developmental features of lexical density in English writings by Chinese beginner learners. In response to this question, the overall level of lexical density in all writing samples was examined first. Then, the developmental trend of lexical density was explored across grade levels.

As shown in Table 5, lexical density is at 41.37, 43.71, and 43.93% in the samples of the three grades. These ratios are lower compared with those in similar studies that focused on intermediate or advanced learners. For instance, the ratio is near 60% in the writings of Chinese English majors’ (Zhu and Wang, 2013; Zheng, 2015) as well as in those of Spanish L2 learners (Schnur, 2021). However, it can be seen that the lexical density of L2 beginners is generally low, indicating the relative simplicity of their writings’ information content. For instance, Fairclough and Belpoliti (2016) found that beginner learners of Spanish manifest a percentage of 46.4% for lexical words in Spanish writing, which is also comparatively low, although a bit higher than the ratios in this study.

**TABLE 5:** Lexical density per grade level.

Grade level	LL	Lexical word tokens	Tokens^5^	Lexical density
7	Grades 7 and 8: LL = −10.62 Sig. (*p*) = 0.00112	6828	16,506	41.37%
8	Grades 8 and 9: LL = −0.09	7205	16,485	43.71%
9	Sig. (*p*) = 0.75805	7167	16,314	43.93%

In terms of the developmental trend of lexical density, Table 5 demonstrates that the ratio of lexical words generally increases with grade level (41.37 < 43.71 < 43.93%). LL shows that the increase is statistically significant from grades 7 to 8 (LL = −10.62) but non-significant from grades 8 to 9 (LL = −0.09). These results indicate that English compositions by beginner learners have higher information load over time, and they thus proceed from a spoken- to a written-like register during 3 years of English learning. However, the progress is non-linear across grade levels. That is, the growth of lexical words accelerates rapidly in grades 7 and 8 but decelerates in grade 9. This positive growth in lexical density parallels the findings from previous studies that took intermediate and advanced learners as participants (Bao, 2008; Wang and Zhou, 2012; Zhu and Wang, 2013; Schnur, 2021).

Furthermore, the distribution of lexical word classes was analyzed across grade levels. As illustrated in Table 6, lexical word classes are unevenly distributed in the samples. The most frequent lexical words used by beginner learners are nouns (approximately 49.2%), which are sequentially followed by verbs (approximately 30.9%), adjectives (approximately 17.3%), and adverbs (approximately 2.5%). A preliminary analysis of the samples reveals that most nouns are indeed grammatically misused by the participants, and they should be converted into other word classes, as shown in Examples (3) and (4) below.

**TABLE 6:** Distribution of lexical word classes across grade levels.

Grade level	Nouns		Verbs		Adjectives		Adverbs	
				
	Frequency/percentage	Normalized frequency	Frequency/percentage	Normalized frequency	Frequency/percentage	Normalized frequency	Frequency/percentage	Normalized frequency
7	3552/52.02%	212.59	1963/28.75%	117.49	1160/16.98%	69.43	153/2.24%	9.16
8	3482/48.32%	208.09	2288/31.76%	136.74	1247/17.31%	74.52	188/2.61%	11.24
9	3389/47.29%	202.62	2322/32.39%	138.83	1256/17.52%	75.09	200/2.79%	11.96
Total	49.2%		30.9%		17.3%		2.5%

(3) At the beginning, I feel very *sorrow*, but now, it is ok now I am used to do it. (070126.txt).

(4) Smoking can *pollution* the places. So we should stop smoking. (080568.txt).

On the one hand, this overreliance on nouns suggests the obstacles that beginner learners encounter in selecting word classes appropriate to the given context. On the other hand, it could also mirror the teacher’s inefficient classroom instruction in changing word classes in class. This result corroborates and complements what was observed by Yoshida (1978), who longitudinally investigated acquisition of English vocabulary by a Japanese child and reported the child’s propensity for using nouns over verbs in the course of the observational period.

In addition, the distribution of lexical word classes varies across grade levels (see Tables 6, 7). The normalized frequency of nouns decreases from grades 7 to 9 (212.59 > 208.09 > 202.62). LL shows that the decreases are non-significant between adjacent grade levels (grades 7 and 8: LL = 0.81; grades 8 and 9: LL = 1.40) but significant between grades 7 and 9 (LL = 4.01). Unlike the downward trend of nouns, the other three classes show upward trends over time (verbs: 117.49 < 136.74 < 138.83; adjectives: 69.437 < 74.52 < 75.09; adverbs: 9.16 < 11.24 < 11.96). LL demonstrates that the increase in verbs is significant from grades 7 to 8 (LL = −24.39) but non-significant from grades 8 to 9 (LL = −0.20). No significant increases were observed between adjacent grade levels in terms of adjectives (grades 7 and 8: LL = −3.02; grades 8 and 9: LL = −0.02) and adverbs (grades 7 and 8: LL = −3.55; grades 8 and 9: LL = −0.35); yet, there was a significant increase in adverbs from grades 7 to 9 (LL = −6.23). Taken together, these results indicate that beginner learners in the upper grades rely on nouns to a lesser extent. Rather, they opt for other word classes to compose L2 writing, i.e., verbs, adjectives, and adverbs. Put differently, there appears to be an improvement in lexical selection and lexical use on the part of beginner learners across grade levels.

**TABLE 7:** Log-likelihood (LL) tests for lexical word classes across grade levels.

Grade levels	Nouns		Verbs		Adjectives		Adverbs	
				
	LL	Sig. (*p*)	LL	Sig. (*p*)	LL	Sig. (*p*)	LL	Sig. (*p*)
7–8	0.81	0.370	−24.39	0.000	−3.02	0.082	−3.55	0.060
8–9	1.40	0.236	−0.20	0.653	−0.02	0.887	−0.35	0.552
7–9	4.01	0.045	−29.73	0.000	−3.71	0.054	−6.23	0.013

In summary, beginner learners generally use few lexical words. During 3 years of English learning, however, there is a non-linear increase in the lexical density across grade levels. In addition, the excessive reliance of beginner learners on nouns reduced with grade level. Learners in the upper grades increased their use of other word classes, including verbs, adjectives, and adverbs.

### Lexical Errors

The fourth research question ascertained whether and, if so, to what extent spelling accuracy increased in English writing by Chinese beginner learners. For this question, we first investigated the overall level of spelling accuracy in beginner learners’ writings. Then, we explored the developmental trend of spelling accuracy across grade levels.

Table 8 presents the frequency of misspellings pertaining to each base list together with their percentages. As shown in the table, words in base list 1 occur 43,254 times, and 440 of them are incorrectly spelled. Misspellings thus account for 1.01% in base list 1. Likewise, words in base lists 2 and 3 appear 3,412 times and 781 times, respectively, and 173 and 94 words, respectively, are misspelled. The percentage of misspellings is therefore 5.07 and 12.04%. A comparison of the data indicates that beginner learners commit the fewest spelling errors with base list 1 (1.01%), and they therefore achieve mastery of the basic vocabulary in the list. This is also true of the vocabulary in base list 2, as misspellings of base list 2 words occur in a relatively low proportion (5.07%). Conversely, the percentage of misspellings rises sharply to 12.04% in terms of sophisticated words in base list 3. The increasing number of misspellings suggests that beginner learners have difficulty memorizing sophisticated word forms, and they fail to fully internalize the orthographic features of advanced vocabulary. Such difficulties might be ascribed to the word length/frequency relationship: the length of a word varies inversely with its frequency in language (Read, 2000; Zhu, 2013; Schmitt, 2014). Accordingly, sophisticated words in the present study are generally longer than basic words, and they pose greater challenges for learners to memorize. This result confirms what was yielded earlier that productive control of sophisticated vocabulary was lacking on the part of beginner learners.

**TABLE 8:** Percentage of misspellings per base list.

Word lists	Frequency of misspellings/total frequency	Percentage
Base list 1	440/43,254	1.01%
Base list 2	173/3412	5.07%
Base list 3	94/781	12.04%

After viewing beginner learners as a whole and comparing the percentage of misspellings per base list, we detected discrepancies between the three grades regarding the total frequency of misspellings. The statistics for each grade level are reported in Table 9. With regard to grades 7 and 8, there is a clear decrease in both misspelling types and tokens. Specifically, misspelling types decrease non-significantly from 159 to 144 (LL = 0.77), whereas misspelling tokens decrease significantly from 272 to 218 (LL = 6.04). However, with regard to grades 8 and 9, misspelling types and tokens vary slightly and non-significantly. Specifically, the error types in the two grades are 144 and 143 (LL = 0.00), and the error tokens are 218 and 217 (LL = 0.00). Taken together, these results suggest that spelling accuracy in the samples increases from grades 7 to 8. With fewer misspellings, compositions written by grade 8 learners transmit information in a more effective manner and therefore ease comprehension by readers. In contrast, no improvement of spelling accuracy was detected in the samples from grades 8 to 9.

**TABLE 9:** Percentage of misspellings per base list and per grade level.

Word lists	Types of misspellings			Tokens of misspellings		
		
	Grade 7	Grade 8	Grade 9	Grade 7	Grade 8	Grade 9
Base list 1	108/664 (16.3%)	80/753 (10.6%)	61/702 (8.7%)	202/15,036 (1.3%)	138/14,636 (0.9%)	100/13,852 (0.7%)
LL	Grades 7 and 8	Grades 8 and 9		Grades 7 and 8	Grades 8 and 9	
	LL = 8.45 Sig. (*p*) = 0.00365	LL = 1.41 Sig. (*p*) = 0.23523		LL = 10.46 Sig. (*p*) = 0.00122	LL = 4.18 Sig. (*p*) = 0.04087	
Base list 2 x	46/275 (16.7%)	44/323 (13.6%)	47/392 (11.9%)	61/901 (6.7%)	53/1059 (5.0%)	59/1452 (4.1%)
LL	Grades 7 and 8	Grades 8 and 9		Grades 7 and 8	Grades 8 and 9	
	LL = 0.95 Sig. (*p*) = 0.33024	LL = 0.37 Sig. (*p*) = 0.54326		LL = 2.60 Sig. (*p*) = 0.10702	LL = 1.21 Sig. (*p*) = 0.27221	
Base list 3	5/86 (5.8%)	20/110 (18.2%)	35/141 (24.8%)	9/171 (5.3%)	27/275 (9.8%)	58/335 (17.3%)
LL	Grades 7 and 8	Grades 8 and 9		Grades 7 and 8	Grades 8 and 9	
	LL = −6.32 Sig. (*p*) = 0.01192	LL = −1.26 Sig. (*p*) = 0.26082		LL = −2.88 Sig. (*p*) = 0.08973	LL = −6.28 Sig. (*p*) = 0.01222	
Total frequency of misspellings LL	159	144	143	272	218	217
	LL = 0.77 Sig. (*p*) = 0.382	LL = 0.00 Sig. (*p*) = 0.956		LL = 6.04 Sig. (*p*) = 0.014	LL = 0.00 Sig. (*p*) = 0.965	

We further compared the percentage of misspellings per word list across grade levels. As can be seen in Table 9, both misspelling types and tokens decline over time in terms of base list 1 (types: 16.3 > 10.6 > 8.7%; tokens: 1.3 > 0.9 > 0.7%). LL demonstrates that the decrease is significant for all adjacent grade levels (LL = 8.45; LL = 10.46; LL = 4.18) but misspelling types between grades 8 and 9 (LL = 1.41). Similarly, the same trend of deceasing is also detected in terms of base list 2 (types: 16.7 > 13.6 > 11.9%; tokens: 6.7 > 5.0 > 4.1%), but none of the decreases are significant between adjacent grade levels (LL = 0.95; LL = 0.37; LL = 2.60; LL = 1.21). In contrast to the gradual decline in the above lists, misspellings in base list 3 show an increase from grades 7 to 9 in either error types or tokens (types: 5.8 < 18.2 < 24.8%; tokens: 5.3 < 9.8 < 17.3%). As regards the types, the increase is significant from grades 7 to 8 (LL = −6.32) but non-significant from grades 8 to 9 (LL = −1.26). As regards tokens, the increase is non-significant from grades 7 to 8 (LL = −2.88) but significant from grades 8 to 9 (LL = −6.28). Taken together, these results indicate that learners in upper grades commit more misspellings of base list 3, as shown in Example (5) below. These misspellings induce the total frequency of spelling errors to remain unchanged in the samples from grades 8 to 9. The above findings demonstrate that beginners can spell high-frequency words more accurately, but low-frequency words are difficult for them to master. In other words, the spelling accuracy of high-frequency words is gradually improved. Specifically, the words in base lists 1 and 2 improve swiftly, while the words in base list 3 develop at a very slow speed.

(5) It *contribles* (<!> contributes) a lot of lung cancer. (090023.txt).

In conclusion, the percentage of misspellings differed from one base list to another: beginner learners committed the least misspellings of base list 1, while they made the most misspellings of base list 3. Regarding the variation of misspellings across grade levels, writing samples from grade 8 contained fewer misspellings than those from grade 7, and they therefore communicate information more effectively. On the contrary, misspellings did not vary from grades 8 to 9. The possible reasons for this discrepancy will be discussed later.

## Discussion

Based on the developmental features of lexical sophistication, lexical variation, lexical density, and lexical errors analyzed in the previous section, we discuss the possible reasons in this section for these developmental features from the perspective of the language exposure hypothesis.

### Lexical Sophistication

As analyzed earlier, Chinese beginner learners are prone to use high-frequency vocabulary in L2 writing, and their productive control of low-frequency words is still at the beginning level. This is in line with the overuse of basic vocabulary characteristic of learners’ L2 writing (Laufer and Nation, 1995; Qin and Wen, 2007; Fairclough and Belpoliti, 2016; Lei and Yang, 2020). Laufer and Nation (1995) suggest that the overwhelming frequency of basic vocabulary characterizes beginner learners’ L2 writing. Additionally, Fairclough and Belpoliti (2016) found that Spanish writings by beginner learners of Spanish comprise a high percentage of basic vocabulary.

In addition, it has been frequently shown that the use of more formal lexical words characterizes native-like writing (Schmitt, 2000). Chinese beginner learners prefer to use high-frequency words, which indicates their low degree of formality in L2 writing. Even advanced, college-level Chinese learners also use numerous informal words in their writings (Qin and Wen, 2007; Lei and Yang, 2020). Thus, the use of formal or advanced words likely takes an extended period of time for learners to progress toward. According to the language exposure hypothesis, L2 acquisition will be promoted by raising the amount of linguistic exposure. Furthermore, as low-frequency words are generally longer, they require more critical mass to acquire. Therefore, extensive exposure to low-frequency words is needed for learners to improve the formality of their writing.

From the analysis in previous section, we can see that the lexical sophistication of Chinese beginner learners increases gradually across grade levels, although generally at a low level. However, the lexical sophistication of Chinese beginners shows unique developmental features compared with previous studies (see Bao, 2008; Laufer and Nation, 1995; Wan, 2010; Zhu and Wang, 2013; González, 2017; Lee et al., 2021; Schnur, 2021); that is, Chinese beginners make progress every year. The upper grades (grades 8–9) develop quickly, and the lower grades (grades 7–8) develop slowly. By contrast, L2 learners in most previous studies made significant progress between each grade level. One possible explanation for this discrepancy is the different vocabulary profiles used in the current study. As introduced earlier, most previous studies have focused on intermediate or advanced language learners and explored the developmental features of lexical sophistication based on LFP. Unlike these studies, the present study makes use of the Beginner Learners’ Writing Vocabulary List, which is more suitable for Chinese beginner learners. This discrepancy probably also contributes to the phases of lexical teaching in Chinese secondary and high schools. Specifically, as high-frequency words are the main focus in English instruction in grades 7 and 8, and as low-frequency words are not explicitly taught, it is extremely difficult for learners to produce low-frequency words regularly. Therefore, the lexical sophistication of the lower grades develops at a comparatively lower speed. However, things are quite different in grade 9. In grade 9, both learners and their schools face the pressure of high school entrance examinations. For one thing, because of the pressure, beginner learners attempt to significantly expand their lexicons by memorizing words excluded from the textbooks in preparation for the examinations. For another, as noted earlier, most junior high schools of China offer more writing classes in grade 9. Learners in grade 9 would be offered more exposure to low-frequency words, which promotes a significant increase in frequency and obvious development of lexical sophistication. The sophisticated vocabulary growth is thus advanced in the third academic year. This also verifies the principal concept of the language exposure hypothesis that linguistic exposure will impact the L2 learning process. In the classroom, the amount of exposure is mostly from the hours of English instruction. With increases to instruction, learners could receive optimal exposure to low-frequency words. When beginner learners’ linguistic exposure reaches critical mass, they are able to make a qualitative leap in using these words.

### Lexical Variation

As analyzed earlier, lexical variation in beginner learners’ writing samples is generally low. A likely explanation for this lexical monotony may be related to the limited exposure to L2 words and the nature of L2 lexical learning. As they are at the initial stage of L2 writing, students’ exposure to various words is far from sufficient. In addition, as lexical learning is primarily receptive (Tracy-Ventura, 2017), beginner learners who are at the initial stage of language acquisition gain vocabulary knowledge more receptively than productively. As a result, they might experience difficulty in producing appropriate lexical items to phrase their ideas, let alone varying lexis properly to rephrase the same content. Therefore, it is very hard for them to use a variety of words in expressing ideas.

Although the lexical variation in beginner learners’ writing samples is generally low, it increases significantly every school year. Though some lexical items recur in the texts of the three grades, learners in the upper grades expand their productive vocabulary repertoire over time, and they purposefully restate the same content with varied related items, such as synonyms, antonyms, and hyponyms. This result is congruent with that of Hsieh (2016), who investigated lexical use in L2 Chinese writing by primary school students. It also coincides with findings from previous studies that used participants at higher proficiency levels (Bao, 2008; Wan, 2010; Wang and Zhou, 2012; Lee et al., 2021; Schnur, 2021). All of the studies showed a significant increase in lexical variation across grade levels.

In addition, learners in lower grades employed diverse basic vocabulary in base list 1, whereas those in grade 9 used varied, sophisticated vocabulary beyond base list 1. According to the language exposure hypothesis, an important way of increasing linguistic exposure is increasing the frequency of linguistic exposure in multiple contexts. The significant increase in lexical variation across grade levels is possibly closely related to the amount of linguistic exposure offered in the classroom. Most teachers will teach new word types according to graded vocabulary lists every year and offer various kinds of activities, such as vocabulary exercises and extracurricular reading, to expose learners to varied words. Therefore, learners could learn and produce more diversified lexical words gradually.

Although the lexical variation of beginner learners in this study increased gradually by year, different grade levels show diverse developmental features. Specifically, grades 7 and 8 have been studying word list 1, so they are more inclined to use words from this list. However, learners in grade 9 have learned and repeatedly encountered more complex words, so they can gradually produce more complicated and diverse words besides words from word list 1.

### Lexical Density

As analyzed earlier, the lexical density of Chinese beginner learners is generally low. It accords with the general cognitive rules of L2 acquisition. As mentioned earlier, lexical density discriminates levels of texts in the oral–written continuum (Halliday, 2002). The less often lexical words are used in a text, the more likely the text is to tend toward a spoken-like register. Accordingly, the comparatively low lexical density in beginner learners’ samples suggests that the informational aspect is less pronounced in their L2 writings, and they show preference for the oral register when structuring their compositions, which further validates the result in the previous section. In other words, formal words tend to be used less frequently, and colloquial words are used more frequently (Schmitt, 2000). According to the language exposure hypothesis, reduced linguistic exposure decelerates L2 acquisition. Due to the overall limited exposure to lexical words in the classroom, lexical density of beginner learners’ writings is generally low, which indicates a spoken-like register. Therefore, extra exposure to lexical words is needed to improve lexical density in writing.

As analyzed earlier, although the lexical density is generally low in Chinese beginner learners’ writings, it increases every school year, significantly from grades 7–8 and slowly from grades 8–9. The yearly increase in lexical density mirrors previous results (Bao, 2008; Zhu and Wang, 2013; Schnur, 2021), although the growth rate varies across group levels. For example, Bao (2008) found that the growth rate at the group level was non-linear, while Zhu and Wang’s (2013) study indicated no significant differences at the group level but a general increase year by year. Language proficiency is one possible explanation for this discrepancy in findings. Unlike previous studies, which mostly focused on intermediate and advanced learners, this study investigates beginner learners. Schmitt (2000, p. 120) points out that “word learning is a complicated but gradual process.” In other words, learners of different proficiency levels show various developmental features in terms of lexical density. This discrepancy may also be related to school curriculum. In grades 8 and 9, function words almost disappear in word lists 2 and 3; learners have been exposed to the bulk of lexical words included in these two word lists. According to the language exposure hypothesis, optimal exposure accelerates the L2 acquisition process. Therefore, as learners in upper grades gain more exposure to complicated lexical words, they can master and produce more lexical words subconsciously. In addition, it is probable that learners in upper grades have reached a plateau in lexical word learning (see Flynn and O’Neil, 1988); thus, the increase in lexical density is slow from grades 8–9.

Furthermore, compared with lower grade learners, the upper grades no longer overuse nouns; instead, they try to use various kinds of lexical words gradually. By observing the corpus, overuse of nouns in the lower grades is due to noun confusion. However, noun confusion in upper grades decreases gradually, and other kinds of word classes increase, demonstrating improved ability in lexical density. This improvement may also be related to the optimal exposure of upper grade learners to lexical words.

### Lexical Errors

As described earlier, the spelling accuracy of the words in base lists and 2 is much higher than that in base list 3. One explanation for this discrepancy is the complexity of the different word lists. It is probably more difficult for beginners to learn words that are more complicated in base list 3. Therefore, learners might need more time to learn and internalize these complex words.

As analyzed earlier, spelling accuracy in the samples increased from grades 7–8, while no improvement was detected in the samples of grades 8 and 9. These results are in line with earlier studies (Engber, 1995; Wan, 2010; Wang and Zhou, 2012; Liu, 2019), even though the current participants are different: while the participants in earlier studies were intermediate or advanced learners who had been learning English for several years, the current participants are Chinese beginner learners of English whose starting ages are 13–16 years old. The beginners show a slightly different feature in the development of spelling ability. While most of the earlier studies found significant declines of lexical errors across grade levels, in this study, we found that beginners have a rapid decline at the beginning stages and a seeming stabilization at the end.

This seeming stabilization raises a question as to whether lexical development temporarily ceases for learners in upper grades. By further examining the relationship between the percentage of misspellings and the number of low-frequency words in the writings of grade 9 students, we found that one important reason might be the rise of low-frequency words in grade 9. The more the learners try to use low-frequency words, the greater the spelling errors they might commit. Therefore, this increase in misspellings may not necessarily denote the cessation of lexical development. Rather, it could contribute to learning benefits of sophisticated vocabulary and interlanguage development. According to Corder (1967), errors reflect the learner’s attempt to build an interlanguage system through the process of hypothesis testing: learners construct hypotheses about the target language and examine them on the evidence available from the language in use. Based on the examination, they revise those that are deviant from the target language norms so that their interlanguage system proceeds progressively to the target language along the continuum. In the present study, the increased misspellings of sophisticated vocabulary results from the fact that learners are lacking in productive knowledge of these words, yet they attempt to grapple with them in L2 writing. This is a process of hypothesis testing in essence. In this process, learners scrutinize their memorization of sophisticated word forms, and their interlanguage system becomes more similar to the target language.

According to the language exposure hypothesis, optimal exposure accelerates L2 acquisition, while reduced exposure produces linguistic duress and slows down the L2 acquisition process. Additionally, complicated words require more critical mass for learners to acquire. From the above discussion, we can see that lexical complexity and learners’ exposure to different base word lists play a decisive role in spelling accuracy. Specifically, high-frequency words are relatively easy and are taught first for a long time, so they can be mastered well in grade 9. Low-frequency words are relatively difficult and are taught later. Thus, learners commit more spelling errors with these words. In order to improve the spelling accuracy of low-frequency words, more exposure to word list 3 is needed.

## Conclusion

This study investigated the developmental features of lexical richness in English compositions by Chinese beginner learners from the perspective of the language exposure hypothesis. The investigation shows that L2 writings produced by beginner learners are comparatively low in lexical richness, incorporating a limited range of vocabulary comprising primarily high-frequency words and function words. The lack of lexical resources reflects the learners’ disposition toward the spoken register in their written products. Furthermore, from a developmental perspective, all four measures yielded significant, albeit non-linear and unevenly paced, developmental changes across the grade levels. The significant lexical growth suggests that L2 writing by beginner learners tends toward a written-like register in the process of English learning.

Based on these findings, there are several pedagogical implications for vocabulary teaching. First, explicit vocabulary instruction for beginner learners should consider informing them about the different lexical features in spoken and written English. Instructional techniques, such as analyses of model texts or contrastive analyses of spoken and written discourses, can make learners aware of the register differences in word choice. Second, since learners were found to use a small number of diversified, sophisticated lexical words and have trouble with the formal aspect of these words, language teachers are advised to encourage learners to take risks by deploying diversified, sophisticated lexical words in L2 writing and to engage them in practice tasks that facilitate learning these words, such as paraphrasing sentences with more diversified or sophisticated lexical words. Additionally, there is a need to introduce word formation rules and spelling rules (see Nation, 2003) in English classes to help learners correct misspellings. Third, the finding that learners mistook a number of nouns for other word classes implies that teachers and learners should move beyond bilingual word list learning and focus on lexical use in context. For instance, English native corpora, such as British National Corpus and Corpus of Contemporary American English, are available teaching resources with which learners can observe the concordance lines of a node word to understand the linguistic context in which the word occurs. Finally, since linguistic exposure frequency is a prerequisite factor for successful L2 acquisition and an effective way to increase linguistic exposure is through additional reading, a variety of reading activities should be provided for learners to expose them to words in varied contexts.

This study makes several noteworthy contributions to L2 research and teaching. First, these findings enhance our understanding of the developmental features of lexical richness in L2 writing between learners with different proficiencies. Second, language exposure has been verified as an important factor in the development of L2 learning. Therefore, the language exposure hypothesis could be adopted as a theoretical framework for analyzing the developmental features of other language systems in learners’ writings, such as the syntactic level, pragmatic level, and discourse level. Finally, this study utilizes a new word list that is different from LFP. It has been confirmed that the new word list is more suitable for beginner learners. Therefore, for one thing, the word list itself is an important contribution to L2 research. For another, these findings could be useful for teachers and researchers to understand the developmental features of lexical richness in L2 writings by beginner learners.

Admittedly, lexical richness does not fully capture L2 writing quality or proficiency. Rather, it is merely one of multiple dimensions, together with grammar, syntax, discourse, accuracy, fluency, etc. Therefore, in further research, we plan to expand this line of research and examine other linguistic features, such as syntactic complexity, in the beginner learner corpus.

## Data Availability Statement

The original contributions generated for this study are included in the article/Supplementary Material, further inquiries can be directed to the corresponding author.

## Ethics Statement

The studies involving human participants were reviewed and approved by Professor Committee of School of Foreign Languages, Northeast Normal University. Written informed consent from the participants’ legal guardian/next of kin was not required to participate in this study in accordance with the national legislation and the institutional requirements.

## Author Contributions

HPZ was responsible for the conception and design of the study, data collection, data analysis and interpretation, writing, developing, and editing the manuscript. MC contributed to the data analysis, interpretation, manuscript development, and editing. XLL contributed to the manuscript development and editing. All authors contributed to the article and approved the submitted version.

## Conflict of Interest

The authors declare that the research was conducted in the absence of any commercial or financial relationships that could be construed as a potential conflict of interest.

## Footnotes

1 In most junior high schools in China, more writing classes are offered in grade 9, normally one period per week.

2 The log-likelihood (LL) test is a common practice in corpus-based second language acquisition studies. It is performed to identify significant differences across the two corpora. Significant levels are defined as follows: Sig. (*p*) = significance (*p*). *p* < 0.05, critical value = 3.84; *p* < 0.01, critical value = 6.63; *p* < 0.001, critical value = 10.83; *p* < 0.0001, critical value = 15.13 (Liang et al., 2010).

3 The text is marked through the Range program according to the base lists each word belongs to. Words in BASEWRD1 are unmarked. Words in BASEWRD2 are marked with <2>. Words in BASEWRD3 are marked with <3>. Off-list words are marked with <!> (Heatley et al., 2002).

4 As the number of tokens is almost the same in the samples of the three grades, comparing the number of types can reflect the differences in TTR across grade levels.

5 The number of vocabulary items coined by beginner learners is excluded in the statistics here.
