# Supplementary material for: Developmental Features of Lexical Richness in English Writings by Chinese Beginner Learners
Source: Front Psychol. 2021 Jun 2;12:665988. doi: 10.3389/fpsyg.2021.665988 (PMC8206476; doi:10.3389/fpsyg.2021.665988)
Supplement: Supplementary file 1 [file Data_Sheet_1.PDF]

Appendix

Table 1: Word List of Each Grade

BASEWRD 1	BASEWRD 2	BASEWRD 3
A 0	ABLE 0	ABILITY 0
AN 0	DISABLED 0	ABILITIES 0
ABOUT 0	ABROAD 0	ABOVE 0
ACROSS 0	ACCEPT 0	ABSENT 0
ACTOR 0	ACCEPTED 0	ACCIDENTAL 0
ACTORS 0	ACCEPTING 0	ACTIVE 0
ACTRESS 0	ACCEPTS 0	ADDRESS 0
ACTRESSES 0	ACCIDENT 0	ADDRESSES 0
AFRAID 0	ACCIDENTS 0	ADMIRE 0
AFRICA 0	ACHIEVE 0	ADMIRING 0
AFTER 0	ACHIEVED 0	ADMIRES 0
AFTERNOON 0	ACHIEVEMENT 0	ADMIRED 0
AFTERNOONS 0	ACHIEVEMENTS 0	ADVANTAGE 0
AGAIN 0	ACHIEVES 0	ADVANTAGES 0
AGE 0	ACHIEVING 0	AFFORD 0
AGES 0	ACT 0	AFFORDED 0
AGO 0	ACTED 0	AFFORDING 0
AIR 0	ACTING 0	AFFORDS 0
ALL 0	ACTION 0	AFRICAN 0
ALONG 0	ACTIONS 0	AFRICANS 0
ALSO 0	ACTS 0	AGREEMENT 0
ALWAYS 0	ACTIVITY 0	AGREEMENTS 0
AMERICAN 0	ACTIVITIES 0	DISAGREEMENTS 0
AMERICANS 0	ACTUALLY 0	DISAGREEMENT 0
AND 0	ADD 0	AHEAD 0
ANIMAL 0	ADDED 0	AIRPORT 0
ANIMALS 0	ADDING 0	AIRPORTS 0
ANOTHER 0	ADDS 0	ALIEN 0
ANSWER 0	ADULT 0	ALIENS 0
ANSWERED 0	ADULTS 0	ALIVE 0
ANSWERING 0	ADVICE 0	ALOUD 0
ANSWERS 0	ADVISE 0	ANCESTOR 0
ANY 0	ADVISING 0	ANCESTORS 0
ANYTHING 0	ADVISES 0	ANNOUNCE 0
APPLE 0	ADVISED 0	ANNOUNCED 0
APPLES 0	AGAINST 0	ANNOUNCES 0
APRIL 0	AGENT 0	ANNOUNCING 0
AROUND 0	AGENTS 0	ANT 0
ARRIVE 0	AGREE 0	ANTS 0

ARRIVED 0
ARRIVES 0
ARRIVING 0
ART 0
ARTS 0
ARTIST 0
ARTISTS 0
ASK 0
ASKED 0
ASKING 0
ASKS 0
AT 0
AUGUST 0
AUNT 0
AUNTS 0
AUSTRALIA 0
AWAY 0
BABY 0
BABIES 0
BACK 0
BAD 0
BADMINTON 0
BADMINTONS 0
BAG 0
BAGS 0
BALL 0
BALLS 0
BANANA 0
BANANAS 0
BANK 0
BANKS 0
BASEBALL 0
BASEBALLS 0
BASKETBALL 0
BASKETBALLS 0
BAT 0
BATS 0
BBC 0
BE 0
AM 0
ARE 0
BEEN 0
IS 0
WAS 0
WERE 0
AGREED 0
AGREEING 0
AGREES 0
DISAGREE 0
DISAGREED 0
DISAGREEING 0
DISAGREES 0
ALARM 0
ALARMS 0
ALLOW 0
ALLOWED 0
ALLOWING 0
ALLOWS 0
ALMOST 0
ALONE 0
ALREADY 0
ALTHOUGH 0
AMAZING 0
AMONG 0
AMUSEMENT 0
AMUSEMENTS 0
ANCIENT 0
ANGRY 0
ANGRIER 0
ANGRIEST 0
ANYONE 0
ANYWAY 0
ANYWHERE 0
ANYMORE 0
APART 0
APARTMENT 0
APARTMENTS 0
APPEAR 0
APPEARED 0
APPEARING 0
APPEARS 0
AREA 0
AREAS 0
ARGUE 0
ARGUING 0
ARGUES 0
ARGUED 0
ARM 0
ARMS 0
ARMY 0
ANYBODY 0
ASIAN 0
ASIANS 0
ATTEND 0
ATTENDS 0
ATTENDING 0
ATTENDED 0
ATTENTION 0
ATTENTIONS 0
AUSTRALIAN 0
AUSTRALIANS 0
AVOID 0
AVOIDS 0
AVOIDING 0
AVOIDED 0
AWFUL 0
BACKGROUND 0
BACKGROUNDS 0
BACKPACK 0
BACKPACKS 0
BADLY 0
BALLOON 0
BALLOONS 0
BANKER 0
BANKERS 0
BASIC 0
BASKET 0
BASKETS 0
BATHROOM 0
BATHROOMS 0
BEAN 0
BEANS 0
BEHAVE 0
BEHAVES 0
BEHAVED 0
BEHAVING 0
BELIEVABLE 0
BELL 0
BELLS 0
BESIDE 0
BESIDES 0
BIN 0
BINS 0
BISCUIT 0
BISCUITS 0

  - BEING 0
- BEACH 0
  - BEACHES 0
- BEAUTIFUL 0
- BECAUSE 0
- BED 0
  - BEDS 0
- BEEF 0
- BEFORE 0
- BEHIND 0
- BEST 0
- BETWEEN 0
- BIG 0
  - BIGGER 0
  - BIGGEST 0
- BIKE 0
  - BIKES 0
- BIRTHDAY 0
  - BIRTHDAYS 0
- BLACK 0
  - BLACKER 0
  - BLACKEST 0
- BLONDE 0
  - BLOND 0
  - BLONDER 0
  - BLONDEST 0
- BLOW 0
  - BLEW 0
  - BLOWING 0
  - BLOWS 0
  - BLOWN 0
- BLUE 0
  - BLUER 0
  - BLUEST 0
- BOAT 0
  - BOATS 0
- BOOK 0
  - BOOKS 0
- BOOKCASE 0
  - BOOKCASES 0
- BORING 0
- BOWL 0
  - BOWLS 0
- BOX 0
  - BOXES 0

  - ARMIES 0
- ARTICLE 0
  - ARTICLES 0
- ASIA 0
- ASLEEP 0
- ASTRONAUT 0
  - ASTRONAUTS 0
- AUTUMN 0
  - AUTUMNS 0
- AVAILABLE 0
- AWAKE 0
- BAMBOO 0
  - BAMBOOS 0
- BAND 0
  - BANDS 0
- BANDAGE 0
  - BANDAGES 0
  - BANDAGED 0
  - BANDAGING 0
- BEAR 0
  - BEARS 0
- BEAT 0
  - BEATS 0
  - BEATING 0
  - BEATEN 0
- BEAUTY 0
  - BEAUTIFULLY 0
  - BEAUTIES 0
- BECOME 0
  - BECAME 0
  - BECOMES 0
  - BECOMING 0
- BEDROOM 0
  - BEDROOMS 0
- BEGIN 0
  - BEGAN 0
  - BEGINNING 0
  - BEGINNINGS 0
  - BEGINS 0
  - BEGUN 0
- BELIEVE 0
  - BELIEVED 0
  - BELIEVES 0
  - BELIEVING 0
  - UNBELIEVABLE 0

- BLACKBOARD 0
  - BLACKBOARDS 0
- BLOCK 0
  - BLOCKS 0
- BLOUSE 0
  - BLOUSES 0
- BOIL 0
  - BOILS 0
  - BOILING 0
  - BOILED 0
- BOOKSTORE 0
  - BOOKSTORES 0
- BORN 0
- BOSS 0
  - BOSSES 0
- BOTTLE 0
  - BOTTLES 0
- BOTTOM 0
  - BOTTOMS 0
- BOW 0
  - BOWS 0
  - BOWING 0
  - BOWED 0
- BRAIN 0
  - BRAINS 0
- BRAND 0
  - BRANDS 0
- BRAZIL 0
- BRITAIN 0
  - BRITISH 0
- BURIAL 0
  - BURIALS 0
- BURN 0
  - BURNED 0
  - BURNING 0
  - BURNS 0
  - BURNT 0
- BUSINESS 0
  - BUSINESSES 0
- CANADIAN 0
  - CANADIANS 0
- CANCEL 0
  - CANCELLED 0
  - CANCELLING 0
  - CANCELS 0

BOY 0	BELONG 0	CAP 0
BOYS 0	BELONGED 0	CAPS 0
BREAD 0	BELONGING 0	CAPITAL 0
BREAKFAST 0	BELONGS 0	CAPITALS 0
BREAKFASTS 0	BELOW 0	CASE 0
BRIDGE 0	BETTER 0	CASES 0
BRIDGES 0	BICYCLE 0	CBA 0
BRING 0	BICYCLES 0	CELEBRATION 0
BRINGING 0	BIRD 0	CELEBRATIONS 0
BRINGS 0	BIRDS 0	CENTRAL 0
BROUGHT 0	BIRTH 0	CEREMONY 0
BROTHER 0	BIRTHS 0	CEREMONIES 0
BROTHERS 0	BIT 0	CHAIN 0
BROWN 0	BLENDER 0	CHAINS 0
BROWNER 0	BLENDERS 0	CHALK 0
BROWNEST 0	BLIND 0	CHANCE 0
BRUSH 0	BLOOD 0	CHANCES 0
BRUSHING 0	BOARD 0	CHEMISTRY 0
BRUSHES 0	BOARDS 0	CHOICE 0
BRUSHED 0	BODY 0	CHOICES 0
BUS 0	BODIES 0	CHOPSTICK 0
BUSES 0	BORED 0	CHOPSTICKS 0
BUSY 0	BORROW 0	CHRISTMAS 0
BUSIEST 0	BORROWS 0	CIRCLE 0
BUSIER 0	BORROWING 0	CIRCLED 0
BUT 0	BORROWED 0	CIRCLES 0
BUTTERFLY 0	BOTH 0	CIRCLING 0
BUTTERFLIES 0	BRAVE 0	CLAY 0
BUY 0	BRAVER 0	CLAYS 0
BOUGHT 0	BRAVEST 0	CLERK 0
BUYING 0	BREAK 0	CLERKS 0
BUYS 0	BREAKING 0	COACH 0
BY 0	BREAKS 0	COACHES 0
BYE 0	BROKE 0	COAL 0
CABBAGE 0	BROKEN 0	COALS 0
CABBAGES 0	BREATHE 0	COAST 0
CAKE 0	BREATHING 0	COASTS 0
CAKES 0	BREATHES 0	COAT 0
CALL 0	BREATHED 0	COATS 0
CALLED 0	BRIGHT 0	COIN 0
CALLING 0	BRIGHTER 0	COINS 0
CALLS 0	BRIGHTEST 0	COLOMBIA 0
CAMP 0	BUILD 0	COMMUNITY 0
CAMPED 0	BUILDING 0	COMMUNITIES 0
CAMPING 0	BUILDINGS 0	COMPETITOR 0

CAMPS 0	BUILDS 0	COMPETITORS 0
CAN 0	BUILT 0	COMPLETE 0
CANADA 0	BUTTER 0	COMPLETED 0
CANDLE 0	CALENDAR 0	COMPLETES 0
CANDLES 0	CALENDARS 0	COMPLETING 0
CANDY 0	CAMERA 0	CONGRATULATE 0
CANDIES 0	CAMERAS 0	CONGRATULATES 0
CAR 0	CANNIBAL 0	CONGRATULATED 0
CARS 0	CANNIBALS 0	CONGRATULATING 0
CARD 0	CARE 0	CONNECT 0
CARDS 0	CARED 0	CONNECTED 0
CARROT 0	CAREFUL 0	CONNECTING 0
CARROTS 0	CAREFULLY 0	CONNECTS 0
CAT 0	CARELESS 0	CONVENIENT 0
CATS 0	CARES 0	CONVERSATION 0
CCTV 0	CARRY 0	CONVERSATIONS 0
CD 0	CARRIED 0	COOKIE 0
CDS 0	CARRIES 0	COOKIES 0
CENTER 0	CARRYING 0	CORNER 0
CENTRE 0	CARTOON 0	CORNERS 0
CENTERS 0	CARTOONS 0	CORRECT 0
CENTRES 0	CATCH 0	COST 0
CHAIR 0	CATCHES 0	COSTING 0
CHAIRS 0	CATCHING 0	COSTS 0
CHEAP 0	CAUGHT 0	COSTUME 0
CHEAPEST 0	CAUSE 0	COSTUMES 0
CHEAPER 0	CAUSED 0	COTTON 0
CHESS 0	CAUSES 0	COTTONS 0
CHICKEN 0	CAUSING 0	COURAGE 0
CHICKENS 0	CELEBRATE 0	COURSE 0
CHILD 0	CELEBRATED 0	COURSES 0
CHILDREN 0	CELEBRATING 0	CREAM 0
CHINA 0	CELEBRATES 0	CREAMS 0
CHINESE 0	CENT 0	CREATE 0
CINEMA 0	CENTS 0	CREATED 0
CINEMAS 0	CENTIMETRE 0	CREATES 0
CLASS 0	CENTIMETER 0	CREATING 0
CLASSES 0	CENTIMETERS 0	CREATIVITY 0
CLASSMATE 0	CENTIMETRES 0	CRISPY 0
CLASSMATES 0	CM 0	CRISPIER 0
CLASSROOM 0	CMS 0	CRISPIEST 0
CLASSROOMS 0	CENTURY 0	CROWD 0
CLEAN 0	CENTURIES 0	CROWDS 0
CLEANED 0	CERTAIN 0	UNCROWDED 0
CLEANER 0	CERTAINLY 0	CRUEL 0

CLEANEST 0	CHALLENGE 0	CRUELLEST 0
CLEANING 0	CHALLENGED 0	CRUELLER 0
CLEANS 0	CHALLENGES 0	CRY 0
CLIMB 0	CHALLENGING 0	CRIED 0
CLIMBS 0	CHANGE 0	CRIES 0
CLIMBING 0	CHANGED 0	CRYING 0
CLIMBED 0	CHANGES 0	CUSTOM 0
CLOCK 0	CHANGING 0	CUSTOMS 0
CLOCKS 0	CHARACTER 0	CUSTOMER 0
CLOTHES 0	CHARACTERS 0	CUSTOMERS 0
CLOUDY 0	CHEAPLY 0	DAILY 0
CLUB 0	CHEAT 0	DARE 0
CLUBS 0	CHEATS 0	DARED 0
COLD 0	CHEATED 0	DARES 0
COLDER 0	CHEATING 0	DARING 0
COLDEST 0	CHECK 0	DEAD 0
COLOR 0	CHECKS 0	DEALING 0
COLOUR 0	CHECKING 0	DEALT 0
COLORS 0	CHECKED 0	DEGREE 0
COLOURS 0	CHEER 0	DEGREES 0
COME 0	CHEERS 0	DESSERT 0
CAME 0	CHEERED 0	DESSERTS 0
COMES 0	CHEERING 0	DIALOG 0
COMING 0	CHEESE 0	DIALOGUES 0
COMPUTER 0	CHEESES 0	DIALOGUE 0
COMPUTERS 0	CHILDHOOD 0	DIALOGS 0
COOKED 0	CHOCOLATE 0	DIRECT 0
COOKING 0	CHOOSE 0	DIRECTOR 0
COOL 0	CHOOSES 0	DIRECTORS 0
COOLEST 0	CHOOSING 0	DIRECTION 0
COOLER 0	CHOSE 0	DIRECTIONS 0
COULD 0	CHOSEN 0	DISAPPEAR 0
COUNTRY 0	CHORE 0	DISAPPEARED 0
COUNTRIES 0	CHORES 0	DISAPPEARING 0
COUNTRYSIDE 0	CLASSIC 0	DISAPPEARS 0
COUSIN 0	CLASSICS 0	DISAPPOINT 0
COUSINS 0	CLEAR 0	DISAPPOINTS 0
COW 0	CLEARED 0	DISAPPOINTING 0
COWS 0	CLEARER 0	DISAPPOINTED 0
CRIME 0	CLEAREST 0	DISBELIEF 0
CRIMES 0	CLEARING 0	DISCOVER 0
CRIMINAL 0	CLEARS 0	DISCOVERED 0
CRIMINALS 0	CLEARLY 0	DISCOVERIES 0
CROSS 0	CLEVER 0	DISCOVERING 0
CROSSED 0	CLEVEREST 0	DISCOVERS 0

CROSSES 0	CLEVERER 0	DISCOVERY 0
CROSSING 0	CLIMBER 0	DIVIDE 0
CROSSINGS 0	CLIMBERS 0	DIVIDED 0
CUP 0	CLOSE 0	DIVIDES 0
CUPS 0	CLOSER 0	DIVIDING 0
CURLY 0	CLOSEST 0	DOCUMENTARY 0
CURLIER 0	CLOUD 0	DOCUMENTARIES 0
CURLIEST 0	CLOUDS 0	DOUBLE 0
CUT 0	COFFEE 0	DOUBLED 0
CUTS 0	COFFEES 0	DOUBLES 0
CUTTING 0	COLLECT 0	DOUBLING 0
CUTE 0	COLLECTS 0	DOUBT 0
DAD 0	COLLECTING 0	DOUBTED 0
DADS 0	COLLECTED 0	DOUBTING 0
DANCE 0	COLLEGE 0	DOUBTS 0
DANCED 0	COLLEGES 0	DRAMA 0
DANCES 0	COMEDY 0	DRAMAS 0
DANCING 0	COMEDIES 0	EARRING 0
DANGER 0	COMFORTABLE 0	EARRINGS 0
DANGERS 0	COMFORTABLY 0	EARTHQUAKE 0
DARK 0	COMMON 0	EARTHQUAKES 0
DARKER 0	COMMONER 0	EAST 0
DARKEST 0	COMMONEST 0	EASTERN 0
DAUGHTER 0	COMMUNICATE 0	EASTER 0
DAUGHTERS 0	COMMUNICATED 0	ECOSYSTEM 0
DAY 0	COMMUNICATES 0	ECOSYSTEMS 0
DAYS 0	COMMUNICATING 0	EDUCATE 0
DEAR 0	COMMUNICATION 0	EDUCATING 0
DEARER 0	COMMUNICATIONS 0	EDUCATES 0
DEAREST 0	COMPARE 0	EDUCATED 0
DECEMBER 0	COMPARED 0	EFFORT 0
DELICIOUS 0	COMPARES 0	EFFORTS 0
DESCRIBE 0	COMPARING 0	ELBOW 0
DESCRIBED 0	COMPETE 0	ELBOWS 0
DESCRIBES 0	COMPETING 0	ELECTRICITY 0
DESCRIBING 0	COMPETES 0	ELECTRONIC 0
DESK 0	COMPETED 0	EMBARRASSED 0
DESKS 0	COMPETITION 0	EMBARRASSING 0
DICTIONARY 0	COMPETITIONS 0	EMPTY 0
DICTIONARIES 0	COMPLETELY 0	ENDING 0
DIFFERENT 0	CONCERT 0	ENDINGS 0
DIFFERENTLY 0	CONCERTS 0	ENEMY 0
DIFFICULT 0	CONDITION 0	ENEMIES 0
DINNER 0	CONDITIONS 0	ENERGY 0
DINNERS 0	CONSIDER 0	ENERGIES 0

- DIRTY 0
  - DIRTIER 0
  - DIRTIEST 0
- DISH 0
  - DISHES 0
- DO 0
  - DID 0
  - DOES 0
  - DOING 0
  - DONE 0
- DOG 0
  - DOGS 0
- DOLLAR 0
  - DOLLARS 0
- DRAGON 0
  - DRAGONS 0
- DRAW 0
  - DRAWING 0
  - DRAWN 0
  - DRAWS 0
  - DREW 0
- DREAM 0
  - DREAMED 0
  - DREAMING 0
  - DREAMS 0
  - DREAMT 0
- DRESS 0
  - DRESSED 0
  - DRESSES 0
  - DRESSING 0
- DRINK 0
  - DRINKS 0
  - DRANK 0
  - DRUNK 0
  - DRINKING 0
- DRUM 0
  - DRUMS 0
- DRY 0
  - DRIEST 0
  - DRIER 0
- DUMPLING 0
  - DUMPLINGS 0
- EACH 0
- EAR 0
  - EARS 0

  - CONSIDERED 0
  - CONSIDERING 0
  - CONSIDERS 0
- CONTINUE 0
  - CONTINUED 0
  - CONTINUES 0
  - CONTINUING 0
- CONTROL 0
  - CONTROLLED 0
  - CONTROLLING 0
  - CONTROLS 0
- COPY 0
  - COPYING 0
  - COPIES 0
  - COPIED 0
- CORN 0
  - CORNS 0
- COUGH 0
  - COUGHS 0
  - COUGHED 0
  - COUGHING 0
- COUNT 0
  - COUNTED 0
  - COUNTING 0
  - COUNTS 0
- COUPLE 0
  - COUPLES 0
- COVER 0
  - COVERED 0
  - COVERING 0
  - COVERS 0
- CRAYON 0
  - CRAYONS 0
- CRAZY 0
  - CRAZIER 0
  - CRAZIEST 0
- CREATIVE 0
- CROWDED 0
- CULTURE 0
  - CULTURES 0
- DANGEROUS 0
- DATE 0
  - DATES 0
- DAYTIME 0
  - DAYTIMES 0

- ENTER 0
  - ENTERED 0
  - ENTERING 0
  - ENTERS 0
- ENVIRONMENTAL 0
- EUROPEAN 0
  - EUROPEANS 0
- EVE 0
  - EVES 0
- EVERYDAY 0
- EXACTLY 0
- EXAMINE 0
  - EXAMINING 0
  - EXAMINES 0
  - EXAMINED 0
  - EXAMINATION 0
  - EXAMINATIONS 0
- EXCEPT 0
- EXCHANGE 0
  - EXCHANGED 0
  - EXCHANGES 0
  - EXCHANGING 0
- EXPRESS 0
  - EXPRESSED 0
  - EXPRESSES 0
  - EXPRESSING 0
  - EXPRESSION 0
  - EXPRESSIONS 0
- FAIL 0
  - FAILED 0
  - FAILING 0
  - FAILS 0
- FAIRS 0
- FAME 0
- FASCINATING 0
- FAULT 0
  - FAULTS 0
- FIELD 0
  - FIELDS 0
- FIN 0
  - FINS 0
- FISHERMAN 0
  - FISHERMEN 0
- FLASH 0
  - FLASHING 0

EARLY 0<br>EARLIER 0<br>EARLIEST 0<br>EASY 0<br>EASIER 0<br>EASIEST 0<br>EASILY 0<br>EAT 0<br>ATE 0<br>EATEN 0<br>EATING 0<br>EATS 0<br>EGG 0<br>EGGS 0<br>EIGHT 0<br>EIGHTEEN 0<br>EIGHTEENTH 0<br>EIGHTH 0<br>EIGHTIETH 0<br>EIGHTY 0<br>EITHER 0<br>ELEPHANT 0<br>ELEPHANTS 0<br>ELEVEN 0<br>ELEVENTH 0<br>E-MAIL 0<br>E-MAILS 0<br>END 0<br>ENDS 0<br>ENGLISH 0<br>ENGLISHES 0<br>ENJOY 0<br>ENJOYED 0<br>ENJOYING 0<br>ENJOYS 0<br>ERASER 0<br>ERASERS 0<br>EUROPE 0<br>EVENING 0<br>EVENINGS 0<br>EVERY 0<br>EVERYTHING 0<br>EVERYWHERE 0<br>EXCELLENT 0<br>EXCITING 0	DEAF 0<br>DEAFER 0<br>DEAFEST 0<br>DEATH 0<br>DEATHS 0<br>DECIDE 0<br>DECIDED 0<br>DECIDES 0<br>DECIDING 0<br>DECISION 0<br>DECISIONS 0<br>DEEP 0<br>DEEPER 0<br>DEEPEST 0<br>DELETE 0<br>DELETES 0<br>DELETED 0<br>DELETING 0<br>DENTIST 0<br>DENTISTS 0<br>DEPEND 0<br>DEPENDS 0<br>DEPENDED 0<br>DEPENDING 0<br>INDEPENDENT 0<br>INDEPENDENCE 0<br>DESERT 0<br>DESERTS 0<br>DEVELOP 0<br>DEVELOPED 0<br>DEVELOPING 0<br>DEVELOPMENT 0<br>DEVELOPMENTS 0<br>DEVELOPS 0<br>DIARY 0<br>DIARIES 0<br>DIE 0<br>DIED 0<br>DIES 0<br>DYING 0<br>DIFFERENCE 0<br>DIFFERENCES 0<br>DIFFICULTY 0<br>DIFFICULTIES 0<br>DIG 0	FLASHES 0<br>FLASHED 0<br>FOLK 0<br>FOOL 0<br>FOOLS 0<br>FOOLING 0<br>FOOLED 0<br>FORK 0<br>FORKS 0<br>FORM 0<br>FORMS 0<br>FRANCE 0<br>FRIDGE 0<br>FRIDGES 0<br>FRIENDLY 0<br>FRIENDSHIP 0<br>GARDEN 0<br>GARDENS 0<br>GATE 0<br>GATES 0<br>GENERAL 0<br>GENERALS 0<br>GENTLEMAN 0<br>GENTLEMEN 0<br>GERMANY 0<br>GHOST 0<br>GHOSTS 0<br>GLASS 0<br>GLOVE 0<br>GLOVES 0<br>GOAL 0<br>GOALS 0<br>GODDESS 0<br>GRADUALLY 0<br>GRADUATE 0<br>GRADUATED 0<br>GRADUATES 0<br>GRADUATING 0<br>GRADUATION 0<br>GRADUATIONS 0<br>GRAMMAR 0<br>GRAMMARS 0<br>GRANDDAUGHTER 0<br>GRANDDAUGHTERS 0<br>GRAPE 0

- EXCUSE 0
    - EXCUSING 0
    - EXCUSES 0
    - EXCUSED 0
- EXERCISE 0
    - EXERCISED 0
    - EXERCISES 0
    - EXERCISING 0
- EXPENSIVE 0
- EYE 0
    - EYES 0
- FACE 0
    - FACES 0
- FAMILY 0
    - FAMILIES 0
- FAR 0
    - FARTHER 0
    - FARTHEST 0
- FARM 0
    - FARMER 0
    - FARMERS 0
    - FARMS 0
    - FARMING 0
    - FARMED 0
- FAST 0
    - FASTER 0
    - FASTEST 0
- FAT 0
    - FATTER 0
    - FATTEST 0
- FATHER 0
    - FATHERS 0
- FAVORITE 0
    - FAVOURITE 0
    - FAVORITES 0
    - FAVOURITES 0
- FEBRUARY 0
- FEED 0
    - FED 0
    - FEEDING 0
    - FEEDS 0
- FEEL 0
    - FEELS 0
    - FELT 0
- FESTIVAL 0
    - DIGGING 0
    - DIGS 0
    - DUG 0
- DISCUSS 0
    - DISCUSSING 0
    - DISCUSSES 0
    - DISCUSSED 0
    - DISCUSSION 0
    - DISCUSSIONS 0
- DISLIKE 0
    - DISLIKES 0
    - DISLIKED 0
    - DISLIKING 0
- DJ 0
    - DJS 0
- DOCTOR 0
    - DOCTORS 0
- DOOR 0
    - DOORS 0
- DRIVER 0
    - DRIVERS 0
- DROP 0
    - DROPPED 0
    - DROPPING 0
    - DROPS 0
- DUCK 0
    - DUCKS 0
- DUE 0
- DURING 0
- EARTH 0
    - EARTHS 0
- EDUCATION 0
    - EDUCATIONAL 0
- ELDER 0
- ELSE 0
- EMPEROR 0
    - EMPERORS 0
- ENCOURAGE 0
    - ENCOURAGING 0
    - ENCOURAGES 0
    - ENCOURAGED 0
- ENDANGERED 0
- ENGINEER 0
    - ENGINEERS 0
- ENGLAND 0
    - GRAPES 0
- GRASS 0
    - GRASSES 0
- GREET 0
    - GREETS 0
    - GREETING 0
    - GREETED 0
- GREY 0
- GUARD 0
    - GUARDED 0
    - GUARDING 0
    - GUARDS 0
- GUY 0
    - GUYS 0
- HALLOWEEN 0
    - HALLOWEENS 0
- HANDBAG 0
    - HANDBAGS 0
- HAPPENINGS 0
- HARMFUL 0
- HAUNTED 0
- HEAT 0
    - HEATED 0
    - HEATING 0
    - HEATS 0
- HEEL 0
    - HEELS 0
- HELPFUL 0
- HERO 0
    - HEROES 0
- HISTORIAN 0
- HISTORICAL 0
- HOAX 0
    - HOAXES 0
- HONOR 0
    - HONOUR 0
    - HONORED 0
    - HONOURED 0
    - HONORING 0
    - HONOURING 0
    - HONORS 0
    - HONOURS 0
- HUG 0
    - HUGGING 0
    - HUGGED 0

FESTIVALS 0<br>FIGHT 0<br>FIGHTING 0<br>FOUGHT 0<br>FIGHTS 0<br>FIND 0<br>FINDING 0<br>FINDS 0<br>FOUND 0<br>FINE 0<br>FINER 0<br>FINEST 0<br>FINISH 0<br>FINISHED 0<br>FINISHES 0<br>FINISHING 0<br>FIRE 0<br>FIRES 0<br>FIRST 0<br>FISH 0<br>FISHES 0<br>FIVE 0<br>FIFTEEN 0<br>FIFTEENTH 0<br>FIFTH 0<br>FIFTIETH 0<br>FIFTY 0<br>FLAG 0<br>FLAGS 0<br>FLOWER 0<br>FLOWERS 0<br>FLY 0<br>FLIES 0<br>FLYING 0<br>FLEW 0<br>FLOWN 0<br>FOLLOW 0<br>FOLLOWED 0<br>FOLLOWING 0<br>FOLLOWS 0<br>FOOD 0<br>FOODS 0<br>FOR 0<br>FOREST 0<br>FORESTS 0	ENJOYABLE 0<br>ENOUGH 0<br>ENVIRONMENT 0<br>ENVIRONMENTS 0<br>EQUATOR 0<br>EQUATORS 0<br>ESPECIALLY 0<br>EVEN 0<br>EVENT 0<br>EVENTS 0<br>EVER 0<br>EVERYBODY 0<br>EVERYONE 0<br>EXAM 0<br>EXAMS 0<br>EXAMPLE 0<br>EXAMPLES 0<br>EXCITE 0<br>EXCITES 0<br>EXCITED 0<br>EXCITEMENT 0<br>EXPECT 0<br>EXPECTED 0<br>EXPECTING 0<br>EXPECTS 0<br>EXPERIENCE 0<br>EXPERIENCES 0<br>EXPERT 0<br>EXPERTS 0<br>EXPLAIN 0<br>EXPLAINED 0<br>EXPLAINING 0<br>EXPLAINS 0<br>FACT 0<br>FACTS 0<br>FACTORY 0<br>FACTORIES 0<br>FAIRNESS 0<br>FALL 0<br>FALLEN 0<br>FALLING 0<br>FALLS 0<br>FELL 0<br>FAMOUS 0<br>FAN 0	HUGS 0<br>HUMOROUS 0<br>IMPOLITE 0<br>INCREASE 0<br>INCREASED 0<br>INCREASES 0<br>INCREASING 0<br>INDUSTRY 0<br>INDUSTRIES 0<br>INEXPENSIVE 0<br>INFLUENCE 0<br>INFLUENCED 0<br>INFLUENCING 0<br>INFLUENCES 0<br>INSECT 0<br>INSECTS 0<br>INSPIRATION 0<br>INSPIRATIONS 0<br>INSTRUCTION 0<br>INSTRUCTIONS 0<br>INSTRUMENT 0<br>INSTRUMENTS 0<br>INTELLIGENT 0<br>INTERNATIONAL 0<br>INTERVIEW 0<br>INTERVIEWED 0<br>INTERVIEWING 0<br>INTERVIEWS 0<br>INTRODUCTION 0<br>INTRODUCTIONS 0<br>IRON 0<br>IRONS 0<br>ITALY 0<br>ITS 0<br>KEYBOARD 0<br>KEYBOARDS 0<br>KICK 0<br>KICKS 0<br>KICKING 0<br>KICKED 0<br>KING 0<br>KINGS 0<br>KISS 0<br>KISSED 0<br>KISSING 0

- FORGET 0
  - FORGETS 0
  - FORGOT 0
  - FORGOTTEN 0
- FOUR 0
  - FORTY 0
  - FOURTEEN 0
  - FOURTEENTH 0
  - FOURTH 0
  - FORTIETH 0
- FREE 0
  - FREER 0
  - FREEST 0
- FRIDAY 0
  - FRIDAYS 0
- FRIEND 0
  - FRIENDS 0
- FROM 0
- FRONT 0
  - FRONTS 0
- FRUIT 0
  - FRUITS 0
- FUN 0
  - FUNNY 0
  - FUNNIEST 0
  - FUNNIER 0
- GAME 0
  - GAMES 0
- GEOGRAPHY 0
- GET 0
  - GETS 0
  - GETTING 0
  - GOT 0
  - GOTTEN 0
- GIFT 0
  - GIFTS 0
- GIRAFFE 0
  - GIRAFFES 0
- GIRL 0
  - GIRLS 0
- GLASSES 0
- GO 0
  - GOES 0
  - GOING 0
  - GONE 0
  - FANS 0
- FANTASTIC 0
- FEAR 0
  - FEARED 0
  - FEARING 0
  - FEARS 0
- FEELINGS 0
- FEVER 0
  - FEVERS 0
- FEW 0
  - FEWER 0
  - FEWEST 0
- FICTION 0
  - FICTIONS 0
- FILL 0
  - FILLED 0
  - FILLING 0
  - FILLS 0
- FILM 0
  - FILMS 0
- FINALLY 0
- FINGER 0
  - FINGERS 0
- FIT 0
  - FITS 0
  - FITTED 0
  - FITTING 0
- FIX 0
  - FIXED 0
  - FIXES 0
  - FIXING 0
- FLASHLIGHT 0
  - FLASHLIGHTS 0
- FLOOR 0
  - FLOORS 0
- FLU 0
  - FLUS 0
- FOLD 0
  - FOLDS 0
  - FOLDING 0
  - FOLDED 0
- FOOT 0
  - FEET 0
- FOOTBALL 0
  - FOOTBALLS 0
  - KISSES 0
- KNOCK 0
  - KNOCKS 0
  - KNOCKING 0
  - KNOCKED 0
- KNOWLEDGE 0
- KOREA 0
- LABORATORY 0
  - LABORATORIES 0
- LADY 0
  - LADIES 0
- LANDED 0
- LANDING 0
- LANTERN 0
  - LANTERNS 0
- LASTLY 0
- LATELY 0
- LAW 0
  - LAWS 0
- LAY 0
  - LAID 0
  - LAYING 0
  - LAYS 0
- LEADER 0
  - LEADERS 0
- LEAF 0
- LEMON 0
  - LEMONS 0
- LEVEL 0
  - LEVELS 0
- LICENSE 0
  - LICENCES 0
  - LICENCE 0
  - LICENSES 0
- LIFELONG 0
- LIFETIME 0
  - LIFETIMES 0
- LIFT 0
  - LIFTED 0
  - LIFTING 0
  - LIFTS 0
- LIST 0
  - LISTS 0
  - LISTED 0
  - LISTING 0

WENT 0
GOOD 0
GRANDFATHER 0
GRANDFATHERS 0
GRANDMA 0
GRANDMAS 0
GRANDMOTHER 0
GRANDMOTHERS 0
GRANDPA 0
GRANDPAS 0
GRANDPARENT 0
GRANDPARENTS 0
GREAT 0
GREATER 0
GREATEST 0
GREEN 0
GREENER 0
GREENEST 0
GROUP 0
GROUPS 0
GROW 0
GREW 0
GROWING 0
GROWN 0
GROWS 0
GUIDE 0
GUIDES 0
GUITAR 0
GUITARS 0
HABIT 0
HABITS 0
HAIR 0
HAIRS 0
HALF 0
HALVES 0
HALL 0
HALLS 0
HALLWAY 0
HALLWAYS 0
HAMBURGER 0
HAMBURGERS 0
BURGER 0
BURGERS 0
HANDSOME 0
HAPPY 0
FORCE 0
FORCES 0
FOREIGN 0
FOREVER 0
FORWARD 0
FORWARDS 0
FORWARDING 0
FORWARDED 0
FOX 0
FOXES 0
FREEZING 0
FRENCH 0
FRESH 0
FRESHER 0
FRESHEST 0
FULL 0
FULLER 0
FULLEST 0
FUTURE 0
FUTURES 0
GERMAN 0
GERMANS 0
GIRLFRIEND 0
GIRLFRIENDS 0
GIVE 0
GAVE 0
GIVEN 0
GIVES 0
GIVING 0
GLAD 0
GLADDER 0
GLADDEST 0
GLUE 0
GOD 0
GODS 0
GOLD 0
GOODBYE 0
GOVERNMENT 0
GOVERNMENTS 0
Grade 0
GradeS 0
GRAVY 0
GRAVIES 0
GROUND 0
GROUNDS 0
LITTER 0
LITTERS 0
LITTERED 0
LITTERING 0
LIVELY 0
LOCAL 0
LOCK 0
LOCKS 0
LOCKING 0
LOCKED 0
LOW 0
LOWER 0
LOWEST 0
LYRICS 0
MAD 0
MADDEST 0
MADDER 0
MAIL 0
MAILS 0
MAILING 0
MAILED 0
MALL 0
MALLS 0
MANAGE 0
MANAGING 0
MANAGES 0
MANAGERS 0
MANAGER 0
MANAGED 0
MANNER 0
MANNERS 0
MARKET 0
MARKETS 0
MARS 0
MASTER 0
MASTERS 0
MASTERED 0
MASTERING 0
MATERIAL 0
MATERIALS 0
MEDICAL 0
MEMORIZE 0
MEMORIZED 0
MEMORIZES 0
MEMORIZING 0

HAPPIER 0	GUESS 0	MENTION 0
HAPPIEST 0	GUESSING 0	MENTIONED 0
HARD 0	GUESSES 0	MENTIONING 0
HARDER 0	GUESSED 0	MENTIONS 0
HARDEST 0	GUEST 0	METAL 0
HAT 0	GUESTS 0	METALS 0
HATS 0	GUN 0	METHOD 0
HAVE 0	GUNS 0	METHODS 0
HAD 0	HALFWAY 0	MEXICO 0
HAS 0	HAND 0	MIDSUMMER 0
HAVING 0	HANDS 0	MINISTER 0
HB 0	HANG 0	MINISTERS 0
HE 0	HANGING 0	MOBILE 0
HIS 0	HANGS 0	MOONCAKE 0
HIM 0	HUNG 0	MOONCAKES 0
HEAD 0	HAPPEN 0	MUSICAL 0
HEADS 0	HAPPENED 0	MYSTERY 0
HEALTHY 0	HAPPENS 0	MYSTERIES 0
HEALTHIER 0	HARDLY 0	NAPKIN 0
HEALTHIEST 0	HARD-WORKING 0	NAPKINS 0
HEAR 0	HATE 0	NATIONAL 0
HEARD 0	HATING 0	NBA 0
HEARING 0	HATES 0	NEARBY 0
HEARS 0	HATED 0	NEARLY 0
HEAVY 0	HEADACHE 0	NOD 0
HEAVIER 0	HEADACHES 0	NODDING 0
HEAVIEST 0	HEADMASTER 0	NODDED 0
HEIGHT 0	HEADMASTERS 0	NODS 0
HEIGHTS 0	HEALTH 0	NOISE 0
HELLO 0	HEART 0	NOISES 0
HELP 0	HEARTS 0	NOON 0
HELPED 0	HEAVILY 0	NOR 0
HELPING 0	HEN 0	NORMALLY 0
HELPS 0	HENS 0	NORTHERN 0
HERE 0	HERSELF 0	NORWAY 0
HI 0	HIDE 0	NOTE 0
HIGH 0	HID 0	NOTES 0
HIGHER 0	HIDDEN 0	NOTING 0
HIGHEST 0	HIDES 0	NOTED 0
HISTORY 0	HIDING 0	NOVEL 0
HISTORIES 0	HILL 0	NOVELS 0
HOME 0	HILLS 0	OFFICER 0
HOMES 0	HIMSELF 0	OFFICERS 0
HOMEWORK 0	HIT 0	OLYMPICS 0
HORSE 0	HITTING 0	OURS 0

HORSES 0	HITS 0	OUTDOORS 0
HOSPITAL 0	HOBBY 0	OVERCOME 0
HOSPITALS 0	HOBBIES 0	OVERCOMING 0
HOST 0	HOLD 0	OVERCOMES 0
HOSTS 0	HELD 0	OVERCAME 0
HOT 0	HOLDING 0	OVERNIGHT 0
HOTTER 0	HOLDS 0	OVERSLEEP 0
HOTTEST 0	HOLE 0	OVERSLEEPS 0
HOTEL 0	HOLES 0	OVERSLEEPING 0
HOTELS 0	HOLIDAY 0	OVERSLEPT 0
HOUR 0	HOLIDAYS 0	PACK 0
HOURS 0	HOMETOWN 0	PACKS 0
HOUSE 0	HOMETOWNS 0	PACKING 0
HOUSES 0	HONEST 0	PACKED 0
HOW 0	HONEY 0	PAIN 0
HUNDRED 0	HONEYS 0	PAINS 0
HUNDREDS 0	HOPE 0	PAINFUL 0
I 0	HOPED 0	PAL 0
ME 0	HOPES 0	PALS 0
MINE 0	HOPING 0	PALACE 0
MY 0	HOUSEWARMING 0	PALACES 0
ICE-CREAM 0	HOUSEWORK 0	PALE 0
ICE-CREAMS 0	HOWEVER 0	PARDON 0
IDEA 0	HUGE 0	PARDONS 0
IDEAS 0	HUMAN 0	PARDONED 0
IF 0	HUMANS 0	PARDONING 0
IMPORTANT 0	HUNGRY 0	PARTNER 0
IN 0	HURRY 0	PARTNERS 0
INDIA 0	HURRIES 0	PASSPORT 0
INTERESTED 0	HURRIED 0	PASSPORTS 0
INTERESTING 0	HURRYING 0	PATIENT 0
INTO 0	HURT 0	PATIENTS 0
IT 0	HURTS 0	PATTERN 0
IVORY 0	HURTING 0	PATTERNS 0
IVORIES 0	HUSBAND 0	PERFORM 0
JACKET 0	HUSBANDS 0	PERFORMS 0
JACKETS 0	ICY 0	PERFORMING 0
JANUARY 0	ILL 0	PERFORMED 0
JEANS 0	ILLNESS 0	PERIOD 0
JOB 0	IMAGINE 0	PERIODS 0
JOBS 0	IMAGINING 0	PHYSICS 0
JOIN 0	IMAGINES 0	PICNIC 0
JOINED 0	IMAGINED 0	PICNICS 0
JOINING 0	IMPORTANCE 0	PIE 0
JOINS 0	IMPROVE 0	PIES 0

JUICE 0	IMPROVING 0	PIERCE 0
JULY 0	IMPROVES 0	PIERCES 0
JUMP 0	IMPROVED 0	PIERCED 0
JUMPS 0	INCLUDE 0	PIERCING 0
JUMPING 0	INCLUDED 0	PINK 0
JUMPED 0	INCLUDES 0	PINKEST 0
JUNE 0	INCLUDING 0	PINKER 0
JUST 0	INDIAN 0	PIONEER 0
KEEP 0	INDIANS 0	PIONEERS 0
KEEPING 0	INFORMATION 0	PITY 0
KEEPS 0	INSIDE 0	PITYING 0
KEPT 0	INSTEAD 0	PITIES 0
KEY 0	INTEREST 0	PITIED 0
KEYS 0	INTERESTS 0	PLASTIC 0
KG 0	INTERNET 0	PLASTICS 0
KILL 0	INTRODUCE 0	PLEASURE 0
KILLS 0	INTRODUCED 0	PLEASURES 0
KILLED 0	INTRODUCES 0	PLENTY 0
KILLING 0	INTRODUCING 0	POEM 0
KILOMETER 0	INVENT 0	POEMS 0
KILOMETRES 0	INVENTS 0	POLICEMAN 0
KILOMETRE 0	INVENTED 0	POLICEMEN 0
KILOMETERS 0	INVENTING 0	POLISH 0
KM 0	INVENTION 0	POLISHING 0
KMS 0	INVENTIONS 0	POLISHES 0
KIND 0	INVITE 0	POLISHED 0
KINDS 0	INVITING 0	POLITE 0
KITCHEN 0	INVITES 0	POLITELY 0
KITCHENS 0	INVITED 0	POLITEST 0
KITE 0	INVITATION 0	POLITER 0
KITES 0	INVITATIONS 0	POPULARITY 0
KNOW 0	ISLAND 0	POSITION 0
KNEW 0	ISLANDS 0	POSITIONS 0
KNOWING 0	ITSELF 0	POSTCARD 0
KNOWN 0	JAPAN 0	POSTCARDS 0
KNOWS 0	JAPANESE 0	POSTMAN 0
KOALA 0	JOKE 0	POSTMEN 0
KOALAS 0	JOKES 0	POUND 0
L 0	JOURNEY 0	POUNDS 0
LAKE 0	JOURNEYS 0	POWER 0
LAKES 0	JOY 0	POWERS 0
LANGUAGE 0	JOYS 0	PRAISE 0
LANGUAGES 0	JUNIOR 0	PRAISES 0
LARGE 0	JUNK 0	PRAISED 0
LARGER 0	KEEPER 0	PRAISING 0

LARGEST 0	KEEPERS 0	PREFER 0
LAST 0	KID 0	PREFERS 0
LATE 0	KIDS 0	PREFERRING 0
LATER 0	KIDDING 0	PREFERRED 0
LATEST 0	KIDDED 0	PRESENT 0
LAZY 0	KILO 0	PRESENTS 0
LAZIER 0	KILOS 0	PRESIDENT 0
LAZIEST 0	KINDNESS 0	PRESIDENTS 0
LEARN 0	KNEE 0	PREVENT 0
LEARNING 0	KNEES 0	PREVENTED 0
LEARNS 0	KNIFE 0	PREVENTING 0
LEARNT 0	KNIVES 0	PREVENTS 0
LEAVE 0	LAUGH 0	PRIDE 0
LEAVING 0	LAUGHS 0	PRIDES 0
LEG 0	LAUGHING 0	PRIME 0
LEGS 0	LAUGHED 0	PRIVATE 0
LESSON 0	LAUGHTER 0	PROCESS 0
LESSONS 0	LEAD 0	PROCESSED 0
LET 0	LEADING 0	PROCESSES 0
LETS 0	LEADS 0	PROCESSING 0
LETTING 0	LED 0	PRODUCE 0
LIBRARY 0	LEAST 0	PRODUCED 0
LIBRARIES 0	LEND 0	PRODUCES 0
LIFE 0	LENDING 0	PRODUCING 0
LIKE 0	LENDS 0	PRODUCT 0
LIKED 0	LENT 0	PRODUCTS 0
LIKES 0	LESS 0	PROFESSIONAL 0
LIKING 0	LETTER 0	PROJECT 0
LION 0	LETTERS 0	PROJECTS 0
LIONS 0	LETTUCE 0	PRONOUNCE 0
LISTEN 0	LETTUCES 0	PRONOUNCING 0
LISTENED 0	LIGHT 0	PRONOUNCES 0
LISTENING 0	LIGHTS 0	PRONOUNCED 0
LISTENS 0	LINE 0	PRONUNCIATION 0
LITTLE 0	LINES 0	PRONUNCIATIONS 0
LIVE 0	LOCATION 0	PROUD 0
LIVED 0	LOCATIONS 0	PROUDEST 0
LIVING 0	LONDON 0	PROUDER 0
LONG 0	LONELY 0	PUBLIC 0
LONGER 0	LOSE 0	PULL 0
LONGEST 0	LOSES 0	PULLED 0
LOOK 0	LOSING 0	PULLING 0
LOOKED 0	LOUD 0	PULLS 0
LOOKING 0	LOUDLY 0	PUNISH 0
LOOKS 0	LOUDEST 0	PUNISHING 0

LOST 0	LOUDER 0	PUNISHES 0
LOT 0	MACHINE 0	PUNISHED 0
LOTS 0	MACHINES 0	PURPOSE 0
LOVE 0	MADAM 0	PURPOSES 0
LOVED 0	MADAMS 0	QUEEN 0
LOVELY 0	MAGAZINE 0	QUEENS 0
LOVELIER 0	MAGAZINES 0	RABBIT 0
LOVELIEST 0	MAGIC 0	RABBITS 0
LOVES 0	MAGICIAN 0	RANG 0
LOVING 0	MAGICIANS 0	RATHER 0
LUCK 0	MAIN 0	RECALL 0
LUCKY 0	MAKER 0	RECALLS 0
LUCKILY 0	MAKERS 0	RECALLED 0
LUCKIEST 0	MALAYSIA 0	RECALLING 0
LUCKIER 0	MALAYSIAN 0	RECEIVE 0
LUNCH 0	MALAYSIANS 0	RECEIVED 0
LUNCHES 0	MAN-MADE 0	RECEIVES 0
M 0	MARK 0	RECEIVING 0
MAKE 0	MARKED 0	RECYCLE 0
MADE 0	MARKING 0	RECYCLES 0
MAKES 0	MARKS 0	RECYCLED 0
MAKING 0	MARRY 0	RECYCLING 0
MAN 0	MARRIED 0	REFLECT 0
MEN 0	MARRIES 0	REFLECTS 0
MANY 0	MARRYING 0	REFLECTING 0
MAP 0	MATCH 0	REFLECTED 0
MAPS 0	MATCHES 0	REGRET 0
MARCH 0	MATTER 0	REGRETTING 0
MATH 0	MATTERS 0	REGRETTED 0
MATHS 0	MAYBE 0	REGRETS 0
MAY 0	MEAL 0	RELATIVE 0
MEAT 0	MEALS 0	RELATIVES 0
MEATS 0	MEAN 0	RELIEF 0
MEDIUM 0	MEANS 0	REMAIN 0
MEET 0	MEANT 0	REMAINED 0
MEETS 0	MEANING 0	REMAINING 0
MET 0	MEANINGLESS 0	REMAINS 0
MESSAGE 0	MEANINGS 0	REPEAT 0
MESSAGES 0	MEDICINE 0	REPEATED 0
MIDDLE 0	MEDICINES 0	REPEATING 0
MILK 0	MEETINGS 0	REPEATS 0
MILKED 0	MEMBER 0	REQUEST 0
MILKS 0	MEMBERS 0	REQUESTS 0
MILKING 0	MEMORY 0	REQUESTING 0
MINUTE 0	MEMORIES 0	REQUESTED 0

MINUTES 0	MENU 0	REQUIRE 0
MISSED 0	MENUS 0	REQUIRED 0
MISSES 0	MESS 0	REQUIRES 0
MISSING 0	MESSES 0	REQUIRING 0
MODEL 0	METER 0	RESPONSIBLE 0
MODELS 0	METRES 0	RESTROOM 0
MOM 0	METRE 0	RESTROOMS 0
MOMS 0	METERS 0	REUSABLE 0
MUM 0	MIDNIGHT 0	REVIEW 0
MUMS 0	MIGHT 0	REVIEWS 0
MONDAY 0	MILE 0	REVIEWED 0
MONDAYS 0	MILES 0	REVIEWING 0
MONEY 0	MILLION 0	RINGING 0
MONKEY 0	MILLIONS 0	ROW 0
MONKEYS 0	MIND 0	ROWS 0
MONTH 0	MINDED 0	RUNG 0
MONTHS 0	MINDING 0	RUSH 0
MOON 0	MINDS 0	RUSHED 0
MORE 0	MIRROR 0	RUSHES 0
MORNING 0	MIRRORS 0	RUSHING 0
MORNINGS 0	MISTAKE 0	SADNESS 0
MOTHER 0	MISTAKES 0	SAFETY 0
MOTHERS 0	MIX 0	SAINT 0
MOUNTAIN 0	MIXING 0	SAINTS 0
MOUNTAINS 0	MIXES 0	SALTY 0
MOUSE 0	MIXED 0	SCIENTIFIC 0
MICE 0	MODERN 0	SCISSORS 0
MOUTH 0	MOONLIGHT 0	SCOOP 0
MOUTHS 0	MOST 0	SCOOPS 0
MOVE 0	MYSELF 0	SCORE 0
MOVED 0	NATURE 0	SCORED 0
MOVES 0	NECESSARY 0	SCORING 0
MOVIE 0	NECK 0	SCORES 0
MOVIES 0	NECKS 0	SEASON 0
MR 0	NEIGHBOR 0	SEASONS 0
MRS 0	NEIGHBORS 0	SECRET 0
MS 0	NEIGHBOUR 0	SECRETS 0
MUCH 0	NEIGHBOURS 0	SELDOM 0
MUSEUM 0	NEITHER 0	SENIOR 0
MUSEUMS 0	NERVOUS 0	SENSE 0
MUSIC 0	NEWS 0	SENSES 0
MUSICIAN 0	NOBODY 0	SENSING 0
MUSICIANS 0	NONE 0	SENSED 0
MUST 0	NORMAL 0	SENTENCE 0
MUTTON 0	NOSEBLEED 0	SENTENCES 0

NAME 0	NOSEBLEEDS 0	SEPARATE 0
NAMES 0	NOTHING 0	SEPARATED 0
NATURAL 0	NOTICE 0	SEPARATES 0
NBA 0	NOTICED 0	SEPARATING 0
NEAR 0	NOTICES 0	SHALL 0
NEED 0	NOTICING 0	SHARK 0
NEEDED 0	NOWADAYS 0	SHARKS 0
NEEDING 0	NURSE 0	SHOULDER 0
NEEDS 0	NURSES 0	SHOULDERS 0
NEIGHBORHOOD 0	OBJECT 0	SHUT 0
NEIGHBORHOODS 0	OBJECTS 0	SHUTTING 0
NEVER 0	OCEAN 0	SHUTS 0
NEW 0	OCEANS 0	SHYNESS 0
NEWER 0	OFF 0	SILENT 0
NEWEST 0	OFFER 0	SILVER 0
NEWSPAPER 0	OFFERED 0	SLEEPY 0
NEWSPAPERS 0	OFFERING 0	SMELL 0
NEXT 0	OFFERS 0	SMELT 0
NICE 0	OIL 0	SMELLS 0
NICER 0	OILS 0	SMELLING 0
NICEST 0	ONCE 0	SMELLED 0
NIGHT 0	ONLINE 0	SMOKE 0
NIGHTS 0	ONTO 0	SMOKED 0
NINE 0	OPEN 0	SMOKES 0
NINETEEN 0	OPENING 0	SMOKING 0
NINETEENTH 0	OPENINGS 0	SMOOTH 0
NINETIETH 0	OPENS 0	SMOOTHER 0
NINETY 0	OPENED 0	SMOOTHEST 0
NINTH 0	OPINION 0	SOCIETY 0
NO 0	OPINIONS 0	SOCIETIES 0
NOISY 0	OPPOSITE 0	SOMEBODY 0
NOISIER 0	ORGANIZE 0	SOUR 0
NOISIEST 0	ORGANISE 0	SOURER 0
NOODLE 0	ORGANISED 0	SOUREST 0
NOODLES 0	ORGANISES 0	SPAGHETTI 0
NORTH 0	ORGANISING 0	SPAGHETTIS 0
NOSE 0	ORGANIZED 0	SPARE 0
NOSES 0	ORGANIZES 0	SPARING 0
NOT 0	ORGANIZING 0	SPARES 0
NOTEBOOK 0	OURSELVES 0	SPARED 0
NOTEBOOKS 0	OUTGOING 0	SPEAKER 0
NOVEMBER 0	OVEN 0	SPEAKERS 0
NOW 0	OVENS 0	SPEECH 0
NUMBER 0	OWN 0	SPEECHES 0
NUMBERS 0	OWNER 0	SPEED 0

- OCTOBER 0
- OF 0
- OFFICE 0
  - OFFICES 0
- OFTEN 0
- OK 0
- OLD 0
  - OLDER 0
  - OLDEST 0
- ON 0
- ONE 0
- ONION 0
  - ONIONS 0
- ONLY 0
- OR 0
- ORANGE 0
  - ORANGES 0
- ORDER 0
  - ORDERED 0
  - ORDERING 0
  - ORDERS 0
- OTHER 0
  - OTHERS 0
- OUT 0
- OUTSIDE 0
- OVER 0
- P 0
- PAINTINGS 0
- PAIR 0
  - PAIRS 0
- PANCAKE 0
  - PANCAKES 0
- PANDA 0
  - PANDAS 0
- PARENT 0
  - PARENTS 0
- PARK 0
  - PARKS 0
- PARTY 0
  - PARTIES 0
- PAST 0
- PAY 0
  - PAID 0
  - PAYING 0
  - PAYS 0
  - OWNERS 0
  - OWNS 0
  - OWNING 0
  - OWNED 0
- PAGE 0
  - PAGES 0
- PAINT 0
  - PAINTED 0
  - PAINTS 0
- PAPER 0
- PARAGLIDING 0
- PART 0
  - PARTED 0
  - PARTING 0
  - PARTS 0
- PART-TIME 0
- PASS 0
  - PASSED 0
  - PASSES 0
  - PASSING 0
- PASSAGE 0
  - PASSAGES 0
- PASSENGER 0
  - PASSENGERS 0
- PEACE 0
  - PEACEFUL 0
- PEEL 0
  - PEELS 0
  - PEELED 0
  - PEELING 0
- PEPPER 0
  - PEPPERS 0
- PERCENT 0
- PERFECT 0
- PERFORMANCE 0
  - PERFORMANCES 0
- PERFORMER 0
  - PERFORMERS 0
- PERHAPS 0
- PERSONAL 0
- PHYSICAL 0
- PIANIST 0
  - PIANISTS 0
- PIECE 0
  - PIECES 0
  - SPEEDS 0
- SPIDER 0
  - SPIDERS 0
- SPREAD 0
  - SPREADING 0
  - SPREADS 0
- STAFF 0
  - STAFFS 0
- STAMP 0
  - STAMPS 0
- STANDARD 0
  - STANDARDS 0
- STARE 0
  - STARED 0
  - STARES 0
  - STARING 0
- STEAL 0
  - STOLE 0
  - STOLEN 0
  - STEALS 0
  - STEALING 0
- STEEL 0
- STICKING 0
- STRANGERS 0
- STUCK 0
- STYLE 0
  - STYLES 0
- SUDDEN 0
- SUGGEST 0
  - SUGGESTED 0
  - SUGGESTING 0
  - SUGGESTION 0
  - SUGGESTIONS 0
  - SUGGESTS 0
- SUIT 0
  - SUITS 0
  - SUITING 0
  - SUITED 0
- SUPERHERO 0
  - SUPERHEROES 0
- SUPPORT 0
  - SUPPORTED 0
  - SUPPORTING 0
  - SUPPORTS 0
- SUPPOSE 0

- PEAR 0
  - PEARS 0
- PEN 0
  - PENS 0
- PENCIL 0
  - PENCILS 0
- PEOPLE 0
- PERSON 0
  - PERSONS 0
- PET 0
  - PETS 0
- PHOTO 0
  - PHOTOS 0
- PIANO 0
  - PIANOS 0
- PICK 0
  - PICKS 0
  - PICKING 0
  - PICKED 0
- PICTURE 0
  - PICTURES 0
- PING-PONG 0
- PLACE 0
  - PLACES 0
- PLANE 0
  - PLANES 0
- PLAY 0
  - PLAYED 0
  - PLAYING 0
  - PLAYS 0
  - PLAYER 0
  - PLAYERS 0
- PLEASE 0
- POLICE 0
- POOL 0
  - POOLS 0
- POPULAR 0
- PORRIDGE 0
- POST 0
- POTATO 0
  - POTATOES 0
- PRACTICE 0
  - PRACTICES 0
  - PRACTICING 0
  - PRACTICED 0
- PIG 0
  - PIGS 0
- PILOT 0
  - PILOTS 0
- PLAN 0
  - PLANNED 0
  - PLANNING 0
  - PLANS 0
- PLANET 0
  - PLANETS 0
- PLANT 0
  - PLANTED 0
  - PLANTING 0
  - PLANTS 0
- PLATE 0
  - PLATES 0
- POINT 0
  - POINTED 0
  - POINTING 0
  - POINTS 0
- POLLUTE 0
  - POLLUTES 0
  - POLLUTED 0
  - POLLUTING 0
  - POLLUTION 0
- POOR 0
  - POORER 0
  - POOREST 0
- POP 0
- POPCORN 0
  - POPCORNS 0
- POPULATION 0
  - POPULATIONS 0
- POSSIBLE 0
- POT 0
  - POTS 0
- POUR 0
  - POURS 0
  - POURING 0
  - POURED 0
- PREDICTION 0
  - PREDICTIONS 0
- PREPARE 0
  - PREPARATION 0
  - PREPARATIONS 0
  - SUPPOSED 0
  - SUPPOSES 0
  - SUPPOSING 0
- SURFACE 0
  - SURFACES 0
- SURVEY 0
  - SURVEYS 0
- SWITZERLAND 0
- TAKEAWAY 0
  - TAKEAWAYS 0
- TASK 0
  - TASKS 0
- TEAMMATE 0
  - TEAMMATES 0
- TEEN 0
  - TEENS 0
  - TEENAGE 0
- TEXT 0
  - TEXTS 0
- TEXTBOOK 0
  - TEXTBOOKS 0
- THANKFUL 0
- THIRSTY 0
  - THIRSTIER 0
  - THIRSTIEST 0
- TIE 0
  - TYING 0
  - TIES 0
  - TIED 0
- TILL 0
- TINY 0
  - TINIER 0
  - TINIEST 0
- TON 0
  - TONS 0
- TOTAL 0
  - TOTALS 0
- TRADE 0
  - TRADED 0
  - TRADES 0
  - TRADING 0
- TRADITION 0
  - TRADITIONS 0
- TRAFFIC 0
  - TRAFFICS 0

PRICE 0	PREPARED 0	TRANSLATE 0
PRICES 0	PREPARES 0	TRANSLATING 0
PROBLEM 0	PREPARING 0	TRANSLATES 0
PROBLEMS 0	PRESS 0	TRANSLATED 0
PURPLE 0	PRESSED 0	TRANSPORTATION 0
PUT 0	PRESSES 0	TRANSPORTATIONS 0
PUTS 0	PRESSING 0	TREAT 0
PUTTING 0	PRESSURE 0	TREATS 0
QUARTER 0	PRESSURES 0	TREATING 0
QUARTERS 0	PRETTY 0	TREATED 0
QUICKLY 0	PRETTIER 0	TRICK 0
QUIET 0	PRETTIEST 0	TRICKS 0
QUIETER 0	PRIMARY 0	TRUCK 0
QUIETEST 0	PRINCE 0	TRUCKS 0
QUILT 0	PRINCES 0	UGLY 0
QUILTS 0	PRINT 0	UGLIEST 0
QUITE 0	PRINTED 0	UGLIER 0
RACE 0	PRINTING 0	UNCOMFORTABLE 0
RACES 0	PRINTS 0	UNDERGROUND 0
RADIO 0	PRIZE 0	UNEASY 0
RADIOS 0	PRIZES 0	UNEXPECTED 0
RAIN 0	PROBABLY 0	VALUE 0
RAINS 0	PROGRESS 0	VALUABLE 0
RAINING 0	PROGRESSED 0	VALUED 0
RAINED 0	PROGRESSES 0	VALUES 0
RAINY 0	PROGRESSING 0	VALUING 0
RAINIER 0	PROMISE 0	VICTORY 0
RAINIEST 0	PROMISED 0	VICTORIES 0
READ 0	PROMISES 0	WAR 0
READING 0	PROMISING 0	WARS 0
READS 0	PROPER 0	WARMTH 0
REAL 0	PROTECT 0	WARN 0
REALLY 0	PROTECTED 0	WARNED 0
RED 0	PROTECTING 0	WARNING 0
REDDER 0	PROTECTION 0	WARNS 0
REDDEST 0	PROTECTIONS 0	WASHROOM 0
RELAX 0	PROTECTS 0	WASHROOMS 0
RELAXES 0	PROVIDE 0	WEALTH 0
RELAXING 0	PROVIDED 0	WEBSITE 0
REMEMBER 0	PROVIDES 0	WEBSITES 0
REMEMBERED 0	PROVIDING 0	WEIGHT 0
REMEMBERING 0	PROVINCE 0	WEST 0
REMEMBERS 0	PROVINCES 0	WHOEVER 0
RESTAURANT 0	PUPIL 0	WHOM 0
RESTAURANTS 0	PUPILS 0	WHOSE 0

- RICE 0
- RIDDEN 0
- RIDING 0
- RIGHT 0
- RIVER 0
  - RIVERS 0
- ROAD 0
  - ROADS 0
- ROBOT 0
  - ROBOTS 0
- RODE 0
- ROOM 0
  - ROOMS 0
- ROPEWAY 0
  - ROPEWAYS 0
- ROUND 0
  - ROUNDER 0
  - ROUNDEST 0
- RULE 0
  - RULES 0
- RUN 0
  - RAN 0
  - RUNNING 0
  - RUNS 0
- RUSSIAN 0
  - RUSSIANS 0
- S 0
- SALAD 0
  - SALADS 0
- SALE 0
  - SALES 0
- SAME 0
- SATURDAY 0
  - SATURDAYS 0
- SAVE 0
  - SAVED 0
  - SAVES 0
  - SAVING 0
- SAY 0
  - SAID 0
  - SAYS 0
- SCARED 0
- SCARY 0
  - SCARIER 0
  - SCARIEST 0
- PUSH 0
  - PUSHING 0
  - PUSHES 0
  - PUSHED 0
- QUESTIONED 0
- QUESTIONING 0
- QUICK 0
  - QUICKEST 0
  - QUICKER 0
- QUIETLY 0
- RAILWAY 0
  - RAILWAYS 0
- RAINSTORM 0
  - RAINSTORMS 0
- RAISE 0
  - RAISED 0
  - RAISES 0
  - RAISING 0
- RAPID 0
- REACH 0
  - REACHED 0
  - REACHES 0
  - REACHING 0
- READY 0
- REALIZE 0
  - REALIZED 0
  - REALIZES 0
  - REALIZING 0
- REASON 0
  - REASONS 0
- RECENTLY 0
- RECORD 0
  - RECORDED 0
  - RECORDING 0
  - RECORDS 0
- REFUSE 0
  - REFUSED 0
  - REFUSES 0
  - REFUSING 0
- REGARD 0
  - REGARDED 0
  - REGARDING 0
  - REGARDS 0
- RELATION 0
  - RELATIONS 0
- WIDELY 0
- WING 0
  - WINGS 0
- WISELY 0
- WOLF 0
  - WOLVES 0
- WOODEN 0
- WORKDAY 0
  - WORKDAYS 0
- WORKER 0
  - WORKERS 0
- WORTH 0
- WOUND 0
  - WOUNDS 0
  - WOUNDED 0
  - WOUNDING 0
- WWF 0
- ZIPPER 0
  - ZIPS 0
  - ZIPPERS 0
  - ZIP 0

- SCHOOL 0
  - SCHOOLS 0
- SCHOOLBAG 0
  - SCHOOLBAGS 0
- SCIENCE 0
  - SCIENCES 0
- SEE 0
  - SAW 0
  - SEEING 0
  - SEEN 0
  - SEES 0
- SELL 0
  - SELLING 0
  - SELLS 0
  - SOLD 0
- SEPTEMBER 0
- SET 0
  - SETS 0
- SEVEN 0
  - SEVENTEEN 0
  - SEVENTEENTH 0
  - SEVENTH 0
  - SEVENTIETH 0
  - SEVENTY 0
- SHE 0
  - HER 0
  - HERS 0
- SHEEP 0
- SHOE 0
  - SHOES 0
- SHOP 0
  - SHOPS 0
  - SHOPPING 0
  - SHOPPED 0
- SHORT 0
  - SHORTER 0
  - SHORTEST 0
  - SHORTS 0
- SHOUT 0
  - SHOUTED 0
  - SHOUTING 0
  - SHOUTS 0
- SHOW 0
  - SHOWED 0
  - SHOWING 0

  - RELATIONSHIP 0
  - RELATIONSHIPS 0
- REMIND 0
  - REMINDS 0
  - REMINDING 0
  - REMINDED 0
- REPAIR 0
  - REPAIRS 0
  - REPAIRING 0
  - REPAIRED 0
- REPLY 0
  - REPLIED 0
  - REPLIES 0
  - REPLYING 0
- REPORT 0
  - REPORTED 0
  - REPORTER 0
  - REPORTERS 0
  - REPORTING 0
  - REPORTS 0
- RESEARCH 0
  - RESEARCHED 0
  - RESEARCHES 0
  - RESEARCHING 0
- RESOLUTION 0
  - RESOLUTIONS 0
- REST 0
  - RESTED 0
  - RESTING 0
  - RESTS 0
- RESULT 0
  - RESULTS 0
- RETURN 0
  - RETURNED 0
  - RETURNING 0
  - RETURNS 0
- RICH 0
  - RICHER 0
  - RICHEST 0
- RISE 0
  - RISES 0
  - RISING 0
  - ROSE 0
  - RISEN 0
- RISK 0

- SHOWN 0
- SHOWS 0

SHOWER 0
  - SHOWERS 0
  - SHOWERING 0
  - SHOWERED 0

SHY 0

SING 0
  - SINGS 0
  - SANG 0
  - SUNG 0
  - SINGER 0
  - SINGERS 0
  - SINGING 0

SISTER 0
  - SISTERS 0

SIT 0
  - SAT 0
  - SITS 0
  - SITTING 0

SIX 0
  - SIXTEEN 0
  - SIXTEENTH 0
  - SIXTH 0
  - SIXTIETH 0
  - SIXTY 0

SIZE 0
  - SIZES 0

SKATE 0
  - SKATES 0
  - SKATED 0
  - SKATING 0

SKIRT 0
  - SKIRTS 0

SLEEP 0
  - SLEEPING 0
  - SLEEPS 0
  - SLEPT 0

SLOW 0
  - SLOWER 0
  - SLOWEST 0

SMALL 0
  - SMALLER 0
  - SMALLEST 0

SMART 0

- RISKS 0
- RISKING 0
- RISKED 0

ROCK 0
  - ROCKS 0

ROCKET 0
  - ROCKETS 0

ROLE 0
  - ROLES 0

RUBBISH 0

SAD 0
  - SADDEST 0
  - SADDER 0

SAFE 0
  - SAFER 0
  - SAFEST 0

SALT 0
  - SALTS 0

SAND 0
  - SANDS 0

SANDWICH 0
  - SANDWICHES 0

SATISFACTION 0

SAYINGS 0

SCARF 0
  - SCARVES 0

SCENE 0
  - SCENES 0

SCHOOLWORK 0

SCIENTIST 0
  - SCIENTISTS 0

SCREEN 0
  - SCREENS 0

SEA 0
  - SEAS 0

SEARCH 0
  - SEARCHING 0
  - SEARCHES 0
  - SEARCHED 0

SEAT 0
  - SEATS 0

SECONDLY 0

SEEM 0
  - SEEMED 0
  - SEEMING 0

SMARTER 0<br>SMARTEST 0<br>SNAKE 0<br>SNAKES 0<br>SNOW 0<br>SNOWS 0<br>SNOWED 0<br>SNOWING 0<br>SNOWY 0<br>SNOWIER 0<br>SNOWIEST 0<br>SNOWMAN 0<br>SNOWMEN 0<br>SO 0<br>SOCCER 0<br>SOCK 0<br>SOCKS 0<br>SOFA 0<br>SOFAS 0<br>SOME 0<br>SOMETIMES 0<br>SON 0<br>SONS 0<br>SOON 0<br>SOONER 0<br>SOONEST 0<br>SORRY 0<br>SORRIEST 0<br>SORRIER 0<br>SOUND 0<br>SOUNDS 0<br>SOUNDED 0<br>SOUNDING 0<br>SOUP 0<br>SOUTH 0<br>SPEAK 0<br>SPEAKING 0<br>SPEAKS 0<br>SPOKE 0<br>SPOKEN 0<br>SPECIAL 0<br>SPECIALS 0<br>SPELL 0<br>SPELT 0<br>SPELLS 0	SEEMS 0<br>SELF-IMPROVEMENT 0<br>SELF-IMPROVEMENTS 0<br>SEND 0<br>SENDING 0<br>SENDS 0<br>SENT 0<br>SERIOUS 0<br>SERIOUSLY 0<br>SERVE 0<br>SERVANT 0<br>SERVANTS 0<br>SERVED 0<br>SERVES 0<br>SERVING 0<br>SERVICE 0<br>SERVICES 0<br>SEVERAL 0<br>SHAKE 0<br>SHAKEN 0<br>SHAKES 0<br>SHAKING 0<br>SHOOK 0<br>SHAME 0<br>SHAPE 0<br>SHAPES 0<br>SHARE 0<br>SHARED 0<br>SHARES 0<br>SHARING 0<br>SHINE 0<br>SHINES 0<br>SHINING 0<br>SHONE 0<br>SHIP 0<br>SHIPS 0<br>SHIRT 0<br>SHIRTS 0<br>SHOCKED 0<br>SHOOT 0<br>SHOOTING 0<br>SHOOTS 0<br>SHOT 0<br>SHOULD 0<br>SICK 0	

SPELLING 0
SPELLED 0
SPEND 0
SPENDING 0
SPENDS 0
SPENT 0
SPORT 0
SPORTS 0
STAR 0
STARS 0
START 0
STARTED 0
STARTING 0
STARTS 0
STATE 0
STATES 0
STATION 0
STATIONS 0
STAY 0
STAYED 0
STAYING 0
STAYS 0
STILL 0
STOP 0
STOPS 0
STORE 0
STORES 0
STORY 0
STORIES 0
STRAIGHT 0
STRAIGHTER 0
STRAIGHTEST 0
STRAWBERRY 0
STRAWBERRIES 0
STREET 0
STREETS 0
STRICT 0
STRICTEST 0
STRICTER 0
STUDENT 0
STUDENTS 0
STUDY 0
STUDIED 0
STUDIES 0
STUDYING 0

SICKEST 0
SICKER 0
SIDE 0
SIDES 0
SIGN 0
SIGNS 0
SILENCE 0
SILK 0
SILKS 0
SILLY 0
SILLIER 0
SILLIEST 0
SIMILAR 0
SIMPLE 0
SIMPLER 0
SIMPLEST 0
SIMPLY 0
SINCE 0
SIR 0
SITCOM 0
SITCOMS 0
SITUATION 0
SITUATIONS 0
SKILL 0
SKILLS 0
SKY 0
SKIES 0
SMILE 0
SMILED 0
SMILES 0
SMILING 0
SNACK 0
SNACKS 0
SOCIAL 0
SOFT 0
SOFTER 0
SOFTEST 0
SOLVE 0
SOLVING 0
SOLVES 0
SOLVED 0
SOMEONE 0
SOMETHING 0
SOMEWHERE 0
SONG 0

- SUBJECT 0
    - SUBJECTS 0
- SUBWAY 0
    - SUBWAYS 0
- SUMMER 0
- SUN 0
    - SUNNY 0
    - SUNNIER 0
    - SUNNIEST 0
- SUNSHINE 0
- SUNDAY 0
    - SUNDAYS 0
- SUPERMARKET 0
    - SUPERMARKETS 0
- SURE 0
    - SURER 0
    - SUREST 0
- SURPRISE 0
    - SURPRISES 0
    - SURPRISING 0
- SWEATER 0
    - SWEATERS 0
- SWIM 0
    - SWUM 0
    - SWIMS 0
    - SWIMMING 0
    - SWAM 0
- SYMBOL 0
    - SYMBOLS 0
- TABLE 0
    - TABLES 0
- TAKE 0
    - TAKEN 0
    - TAKES 0
    - TAKING 0
    - TOOK 0
- TALK 0
    - TALKED 0
    - TALKING 0
    - TALKS 0
- TALL 0
    - TALLEST 0
    - TALLER 0
- TAPE 0
    - TAPES 0

    - SONGS 0
- SORE 0
    - SORER 0
    - SOREST 0
- SOUTHERN 0
- SOUTHWESTERN 0
- SPACE 0
    - SPACES 0
- SPIRIT 0
    - SPIRITS 0
- SPOON 0
    - SPOONS 0
- SPRING 0
- SQUARE 0
    - SQUARES 0
- STAND 0
    - STANDING 0
    - STANDS 0
    - STOOD 0
- STEP 0
    - STEPS 0
- STEPMOTHER 0
    - STEPMOTHERS 0
- STEPSISTER 0
    - STEPSISTERS 0
- STOMACH 0
    - STOMACHS 0
    - STOMACHACHE 0
    - STOMACHACHES 0
- STONE 0
    - STONES 0
- STORM 0
    - STORMS 0
- STRANGE 0
    - STRANGEST 0
- STRESS 0
- STRONG 0
    - STRONGER 0
    - STRONGEST 0
- STUPID 0
    - STUPIDEST 0
    - STUPIDER 0
- SUCCEED 0
    - SUCCEEDED 0
    - SUCCEEDING 0

TASTE 0	SUCCEEDS 0	
TASTED 0	SUCCESS 0	
TASTES 0	SUCCESSES 0	
TASTING 0	SUCCESSFUL 0	
TEA 0	SUCH 0	
TEACH 0	SUDDENLY 0	
TAUGHT 0	SUGAR 0	
TEACHER 0	SUGARS 0	
TEACHERS 0	SUNBURNED 0	
TEACHES 0	SWEEP 0	
TEACHING 0	SWEPT 0	
TELEPHONE 0	SWEEPS 0	
TELEPHONES 0	SWEEPING 0	
PHONES 0	SWEET 0	
PHONE 0	SWEETER 0	
TELL 0	SWEETEST 0	
TELLING 0	SWING 0	
TELLS 0	SWUNG 0	
TOLD 0	SWINGS 0	
TEN 0	SWINGING 0	
TENTH 0	TAIL 0	
TENNIS 0	TAILS 0	
TENT 0	TALENT 0	
TENTS 0	TALENTED 0	
TERM 0	TALENTS 0	
TERMS 0	TAXI 0	
TERRIBLE 0	TAXIS 0	
TEST 0	TEAM 0	
TESTS 0	TEAMS 0	
THAI 0	TECHNOLOGY 0	
THAIS 0	TECHNOLOGIES 0	
THAILAND 0	TEENAGER 0	
THANK 0	TEENAGERS 0	
THANKS 0	TELEVISION 0	
THANKING 0	TELEVISIONS 0	
THANKED 0	TEMPERATURE 0	
THAT 0	TEMPERATURES 0	
THOSE 0	TERRORIST 0	
THE 0	TERRORISTS 0	
THEN 0	THAN 0	
THERE 0	THANKSGIVING 0	
THEY 0	THEATER 0	
THEIR 0	THEATRES 0	
THEM 0	THEATRE 0	
THIN 0	THEATERS 0	

THINNEST 0<br>THINNER 0<br>THING 0<br>THINGS 0<br>THINK 0<br>THINKING 0<br>THINKS 0<br>THOUGHT 0<br>THIS 0<br>THESE 0<br>THREE 0<br>THIRD 0<br>THIRTEEN 0<br>THIRTEENTH 0<br>THIRTY 0<br>THIRTIETH 0<br>THURSDAY 0<br>THURSDAYS 0<br>TIDY 0<br>TIDIER 0<br>TIDIEST 0<br>TIGER 0<br>TIGERS 0<br>TIME 0<br>TIMES 0<br>TIRED 0<br>TO 0<br>TODAY 0<br>TOFU 0<br>TOMATO 0<br>TOMATOES 0<br>TOMORROW 0<br>TOMORROWS 0<br>TONIGHT 0<br>TOO 0<br>TOOTH 0<br>TEETH 0<br>TOWN 0<br>TOWNS 0<br>TREE 0<br>TREES 0<br>TRIP 0<br>TRIPS 0<br>TROUSERS 0<br>TRUE 0	THEME 0<br>THEMES 0<br>THEMSELVES 0<br>THICK 0<br>THICKEST 0<br>THICKER 0<br>THOUGH 0<br>THOUSAND 0<br>THOUSANDS 0<br>THROAT 0<br>THROATS 0<br>THROUGH 0<br>THROW 0<br>THREW 0<br>THROWING 0<br>THROWN 0<br>THROWS 0<br>TICKET 0<br>TICKETS 0<br>TOGETHER 0<br>TOILET 0<br>TOILETS 0<br>TOOL 0<br>TOOLS 0<br>TOOTHACHE 0<br>TOOTHACHES 0<br>TOP 0<br>TOPS 0<br>TOUCH 0<br>TOUCHED 0<br>TOUCHES 0<br>TOUCHING 0<br>TOUR 0<br>TOURS 0<br>TOURING 0<br>TOURED 0<br>TOURIST 0<br>TOURISTS 0<br>TOWARDS 0<br>TOWER 0<br>TOWERS 0<br>TOY 0<br>TOYS 0<br>TRADER 0<br>TRADERS 0	

TRUEST 0<br>TRUER 0<br>T-SHIRT 0<br>T-SHIRTS 0<br>TUESDAY 0<br>TUESDAYS 0<br>TURN 0<br>TURNED 0<br>TURNING 0<br>TURNS 0<br>TV 0<br>TVS 0<br>TWO 0<br>SECOND 0<br>TWELVE 0<br>TWELFTH 0<br>TWENTY 0<br>TWENTIETH 0<br>UFO 0<br>UFOS 0<br>UNCLE 0<br>UNCLES 0<br>UNDER 0<br>UNIFORM 0<br>UNIFORMS 0<br>UP 0<br>USE 0<br>USED 0<br>USEFUL 0<br>USES 0<br>USING 0<br>USUALLY 0<br>VACATION 0<br>VACATIONS 0<br>VEGETABLE 0<br>VEGETABLES 0<br>VERY 0<br>VILLAGE 0<br>VILLAGER 0<br>VILLAGERS 0<br>VILLAGES 0<br>VIOLIN 0<br>VIOLINS 0<br>VISIT 0<br>VISITED 0	TRADITIONAL 0<br>TRAINED 0<br>TRAINING 0<br>TRAININGS 0<br>TRAVEL 0<br>TRAVELED 0<br>TRAVELING 0<br>TRAVELLED 0<br>TRAVELLER 0<br>TRAVELLERS 0<br>TRAVELLING 0<br>TRAVELS 0<br>TRAVELER 0<br>TRAVELERS 0<br>TREASURE 0<br>TREASURES 0<br>TROUBLE 0<br>TROUBLES 0<br>TRULY 0<br>TRUST 0<br>TRUSTED 0<br>TRUSTING 0<br>TRUSTS 0<br>TRUTH 0<br>TRUTHFUL 0<br>TRY 0<br>TRIED 0<br>TRIES 0<br>TRYING 0<br>TURKEY 0<br>TURKEYS 0<br>TWICE 0<br>TYPICAL 0<br>UMBRELLA 0<br>UMBRELLAS 0<br>UNDERSTAND 0<br>UNDERSTANDING 0<br>UNDERSTANDS 0<br>UNDERSTOOD 0<br>UNDERWEAR 0<br>UNDERWEARS 0<br>UNFAIR 0<br>UNIVERSITY 0<br>UNIVERSITIES 0<br>UNLESS 0	

- VISITING 0
    - VISITOR 0
    - VISITORS 0
    - VISITS 0
- VOLLEYBALL 0
    - VOLLEYBALLS 0
- WAKE 0
    - WOKE 0
    - WAKING 0
    - WAKES 0
    - WOKEN 0
- WALK 0
    - WALKED 0
    - WALKING 0
    - WALKS 0
- WANT 0
    - WANTED 0
    - WANTING 0
    - WANTS 0
- WARM 0
    - WARMER 0
    - WARMEST 0
- WASH 0
    - WASHED 0
    - WASHES 0
    - WASHING 0
- WATCH 0
    - WATCHED 0
    - WATCHES 0
    - WATCHING 0
- WATER 0
- WAY 0
    - WAYS 0
- WE 0
    - OUR 0
    - US 0
- WEAR 0
    - WEARING 0
    - WEARS 0
    - WORE 0
    - WORN 0
- WEATHER 0
- WEDNESDAY 0
    - WEDNESDAYS 0
- WEEK 0

- UNLUCKY 0
    - UNLUCKIEST 0
    - UNLUCKIER 0
- UNTIL 0
- UPSET 0
- USUAL 0
    - UNUSUAL 0
- VIDEO 0
    - VIDEOS 0
- VIOLINIST 0
    - VIOLINISTS 0
- VOICE 0
    - VOICES 0
- VOLUNTEER 0
    - VOLUNTEERING 0
    - VOLUNTEERED 0
    - VOLUNTEERS 0
- WAIT 0
    - WAITED 0
    - WAITING 0
    - WAITS 0
- WALL 0
    - WALLS 0
- WALLET 0
    - WALLETS 0
- WASTE 0
    - WASTED 0
    - WASTES 0
    - WASTING 0
- WATERMELON 0
    - WATERMELONS 0
- WEAK 0
    - WEAKER 0
    - WEAKEST 0
- WEEKDAY 0
    - WEEKDAYS 0
- WEEKLY 0
- WESTERN 0
- WET 0
    - WETTER 0
    - WETTEST 0
- WHALE 0
    - WHALES 0
- WHATEVER 0
- WHEEL 0

WEEKS 0	WHEELS 0	
WEEKEND 0	WHENEVER 0	
WEEKENDS 0	WHETHER 0	
WELCOME 0	WHICH 0	
WELL 0	WHILE 0	
WHAT 0	WHOLE 0	
WHEN 0	WIDE 0	
WHERE 0	WIDER 0	
WHITE 0	WIDEST 0	
WHITER 0	WIFE 0	
WHITEST 0	WIVES 0	
WHO 0	WILD 0	
WHY 0	WILDER 0	
WILL 0	WILDEST 0	
WINDY 0	WIN 0	
WINTER 0	WINNER 0	
WINTERS 0	WINNERS 0	
WISH 0	WINNING 0	
WISHED 0	WINS 0	
WISHES 0	WON 0	
WISHING 0	WIND 0	
WITH 0	WINDOW 0	
WOMAN 0	WINDOWS 0	
WOMEN 0	WITHOUT 0	
WOOF 0	WONDER 0	
WORKED 0	WONDERED 0	
WORKING 0	WONDERFUL 0	
WORLD 0	WONDERING 0	
WORLDS 0	WONDERS 0	
WORRY 0	WOOD 0	
WORRYING 0	WOODS 0	
WORRIES 0	WORD 0	
WORRIED 0	WORDS 0	
WOULD 0	WORSE 0	
WOW 0	WORST 0	
WRITE 0	WRITER 0	
WRITES 0	WRITERS 0	
WRITING 0	WRONG 0	
WRITTEN 0	X-RAY 0	
WROTE 0	X-RAYS 0	
YEAH 0	YARD 0	
YEAR 0	YARDS 0	
YEARS 0	YOGURT 0	
YELLOW 0	YOGURTS 0	
YELLOWER 0	YOGHURT 0	

YELLOWEST 0<br>YES 0<br>YESTERDAY 0<br>YET 0<br>YOU 0<br>YOUR 0<br>YOURS 0<br>YOUNG 0<br>YOUNGER 0<br>YOUNGEST 0<br>ZERO 0<br>ZOO 0<br>ZOOS 0	YOGHURTS 0<br>YOURSELF 0<br>YOURSELVES 0	

Table 2: Words with Both British English Form and American English Form

BASEWRD 1	BASEWRD 2	BASEWRD 3
center<br>centre<br>centers<br>centres<br>color<br>colour<br>colors<br>colours<br>favorite<br>favourite<br>favorites<br>favourites<br>kilometer<br>kilometres<br>kilometre<br>kilometers<br>km<br>kms<br>math<br>maths<br>mom<br>moms	centimetre<br>centimeter<br>centimeters<br>centimetres<br>cm<br>cms<br>neighbor<br>neighbors<br>neighbour<br>neighbours<br>organize<br>organise<br>organised<br>organises<br>organising<br>organized<br>organizes<br>organizing<br>theater<br>theatres<br>theatre<br>theaters	dialog<br>dialogues<br>dialogue<br>dialogs<br>honor<br>honour<br>honored<br>honoured<br>honoring<br>honouring<br>honors<br>honours<br>license<br>licences<br>licence<br>licenses

mum<br>mums	yogurt<br>yogurts<br>yoghurt<br>yoghurts	

Table 3：Words with Both Full Forms and Abbreviations

BASEWRD 1	BASEWRD 2	BASEWRD 3
kilometer<br>kilometres<br>kilometre<br>kilometers<br>km<br>kms	centimetre<br>centimeter<br>centimeters<br>centimetres<br>cm<br>cms	

Table 4：Different Compound Words Containing a Same Part

BASEWRD 1	BASEWRD 2	BASEWRD 3
any<br>anything<br>every<br>everything<br>everywhere<br>grandfather<br>grandfathers<br>grandma<br>grandmas<br>grandmother<br>grandmothers<br>grandpa<br>grandpas<br>grandparent<br>grandparents<br>out<br>outside<br>snow<br>snows<br>snowed	anyone<br>anyway<br>anywhere<br>anymore<br>everybody<br>everyone<br>foot<br>feet<br>football<br>footballs<br>pop<br>popcorn<br>popcorns<br>someone<br>something<br>somewhere<br>stepmother<br>stepmothers<br>stepsister<br>stepsisters	lifelong<br>lifetime<br>lifetimes<br>postcard<br>postcards<br>postman<br>postmen<br>text<br>texts<br>textbook<br>textbooks

snowing		
snowy		
snowier		
snowiest		
snowman		
snowmen		
some		
sometimes		
sun		
sunny		
sunnier		
sunniest		
sunshine		
week		
weeks		
weekend		
weekends		
class		
classes		
classmate		
classmates		
classroom		
classrooms		

Table 5：Words with Both Complete Forms and Short Forms

BASEWRD 1	BASEWRD 2	BASEWRD 3
hamburger		zipper
hamburgers		zips
burger		zippers
burgers		zip
telephone		
telephones		
phones		
phone		

Table 6: Words in the Same Word Family but Distributed in Different Word Lists

BASEWRD 1	BASEWRD 2	BASEWRD 3
actor<br>actors<br>actress<br>actresses	act<br>acted<br>acting<br>action<br>actions<br>acts	
	accident<br>accidents	accidental
	activity<br>activities	active
	agree<br>agreed<br>agreeing<br>agrees<br>disagree<br>disagreed<br>disagreeing<br>disagrees	agreement<br>agreements<br>disagreements<br>disagreement
australia		australian<br>australians
beautiful	beauty<br>beautifully<br>beauties	
	believe<br>believed<br>believes<br>believing<br>unbelievable	believable<br>disbelief
canada		canadian<br>canadians
	care<br>cared<br>careful<br>carefully	caring

	careless	
	cares	
	celebrate	celebration
	celebrated	celebrations
	celebrating	
	celebrates	
cheap	cheaply	
cheapest		
cheaper		
climb	climber	
climbs	climbers	
climbing		
climbed		
	compete	competitor
	competing	competitors
	competes	
	competed	
	competition	
	competitions	
	completely	complete
		completed
		completes
		completing
	creative	create
		created
		creates
		creating
		creativity
	crowded	crowd
		crowds
		uncrowded
danger	dangerous	
dangers	endangered	
different	difference	
differently	differences	
difficult	difficulty	

	difficulties	
	education	educate
	educational	educating
		educates
		educated
	environment	environmental
	environments	
europe		european
		europeans
exciting	excite	
	excites	
	excited	
	excitement	
feel	feeling	
feels	feelings	
felt		
fish		fisherman
fishes		fishermen
	french	france
friend		friendly
friends		friendship
glasses		glass
	german	germany
	germans	
	god	goddess
	gods	
	happen	happening
	happened	happenings
	happens	
he	himself	
his		
him		
healthy	health	
healthier		
healthiest		
heavy	heavily	

heavier		
heaviest		
help		helpful
helped		
helping		
helps		
history		historian
histories		historical
i	myself	
me		
mine		
my		
important	importance	
india	indian	
	indians	
interested	interest	
interesting	interests	
	introduce	introduction
	introduced	introductions
	introduces	
	introducing	
it	itself	its
keep	keeper	
keeping	keepers	
keeps		
kept		
know		knowledge
knew		
knowing		
known		
knows		
last		lastly
late		lately
later		
latest		
	lead	leader

	leading	leaders
	leads	
	led	
little	least	
	less	
like	dislike	
liked	dislikes	
likes	disliked	
liking	disliking	
live		lively
lived		
living		
	location	local
	locations	
luck	unlucky	
lucky	unluckiest	
luckily	unluckier	
luckiest		
luckier		
make	maker	
made	makers	
makes		
making		
	medicine	medical
	medicines	
	memory	memorize
	memories	memorized
		memorizes
		memorizing
move		moving
moved		
moves		
music		musical
musician		
musicians		
near		nearby

		nearly
neighborhood	neighbor	
neighborhoods	neighbors	
	neighbour	
	neighbours	
night	midnight	
nights		
noisy		noise
noisier		noises
noisiest		
	normal	normally
north		northern
now	nowadays	
office		officer
offices		officers
painting	paint	
paintings	painted	
	paints	
	performance	
	performances	
	performer	perform
	performers	performs
		performing
		performed
person	personal	
persons		
piano	pianist	
pianos	pianists	
popular		popularity
quickly	quick	
	quickest	
	quicker	
quiet	quietly	
quieter		
quietest		
relax		relaxed

relaxes		
relaxing		
	sad	sadness
	saddest	
	sadder	
	safe	safety
	safer	
	safest	
	salt	salty
	salts	
say	saying	
said	sayings	
says		
science	scientist	scientific
sciences	scientists	
she	herself	
her		
hers		
shy		shyness
	silence	silent
sleep		sleepy
sleeping		
sleeps		
slept		
	social	society
		societies
south	southern	
	southwestern	
speak		speaker
speaking		speakers
speaks		
spoke		
spoken		
summer		midsummer
	strange	stranger
	strangest	strangers

surprise	surprised	
surprises		
surprising		
	suddenly	sudden
	teenager	teen
	teenagers	teens
		teenage
thank		thankful
thanks		
thanking		
thanked		
they	themselves	
their		
them		
tooth	toothache	
teeth	toothaches	
	trader	trade
	traders	traded
		trades
		trading
	traditional	tradition
		traditions
true	truly	
truest	truth	
truer	truthful	
two	twice	
second		
twelve		
twelfth		
twenty		
twentieth		
usually	usual	
	unusual	
	comfortable	uncomfortable
	comfortably	
easy		uneasy

easier		
easiest		
easily		
	expect	unexpected
	expected	
	expecting	
	expects	
violin	violinist	
violins	violinists	
warm		warmth
warmer		
warmest		
we	ourselves	ours
our		
us		
week	weekly	
weeks		
	western	west
who		whom
		whose
	wide	widely
	wider	
	widest	
windy	wind	
	wood	wooden
	woods	
bad	worse	badly
	worst	
write	writer	
writes	writers	
writing		
written		
wrote		
you	yourself	
your	yourselves	
yours		

use		reusable
used		
useful		
uses		
using		

Table 7: Derivations in Word Families

BASEWRD 1	BASEWRD 2	BASEWRD 3
art	achieve	agreement
arts	achieved	agreements
artist	achievement	disagreements
artists	achievements	disagreement
china	achieves	attend
chinese	achieving	attends
cross	act	attending
crossed	acted	attended
crosses	acting	attention
crossing	action	attentions
crossings	actions	britain
different	acts	british
differently	agree	create
easy	agreed	created
easier	agreeing	creates
easiest	agrees	creating
easily	disagree	creativity
eight	disagreed	crowd
eighteen	disagreeing	crowds
eighteenth	disagrees	uncrowded
eighth	beauty	discover
eightieth	beautifully	discovered
eighty	beauties	discoveries
eleven	begin	discovering
eleventh	began	discovers
farm	beginning	discovery
farmer	beginnings	east
farmers	begins	eastern

farms	begun	examine
farming	believe	examining
farmed	believed	examines
five	believes	examined
fifteen	believing	examination
fifteenth	unbelievable	examinations
fifth	build	express
fiftieth	building	expressed
fifty	buildings	expresses
four	builds	expressing
forty	built	expression
fourteen	care	expressions
fourteenth	cared	graduate
fourth	careful	graduated
fortieth	carefully	graduates
fun	careless	graduating
funny	cares	graduation
funniest	clear	graduations
funnier	cleared	manage
love	clearer	managing
loved	clearest	manages
lovely	clearing	managers
lovelier	clears	manager
loveliest	clearly	managed
loves	comfortable	pain
loving	comfortably	pains
luck	communicate	painful
lucky	communicated	polite
luckily	communicates	politely
luckiest	communicating	politest
luckier	communication	politer
music	communications	produce
musician	compete	produced
musicians	competing	produces
play	competes	producing
played	competed	product

	playing		competition		products
	plays		competitions	pronounce	
	player	decide			pronouncing
	players		decided		pronounces
rain			decides		pronounced
	rains		deciding		pronunciation
	raining		decision		pronunciations
	rained		decisions	suggest	
	rainy	depend			suggested
	rainier		depends		suggesting
	rainiest		depended		suggestion
real			depending		suggestions
	really		independent		suggests
seven			independence	teen	
	seventeen	develop			teens
	seventeenth		developed		teenage
	seventh		developing	value	
	seventieth		development		valuable
	seventy		developments		valued
short			develops		values
	shorter	discuss			valuing
	shortest		discussing		
	shorts		discusses		
sing			discussed		
	sings		discussion		
	sang		discussions		
	sung	education			
	singer		educational		
	singers	excite			
	singing		excites		
snow			excited		
	snows		excitement		
	snowed	fair			
	snowing		fairer		
	snowy		fairest		
	snowier		fairness		

snowiest	unfair	
sun	ill	
sunny	illness	
sunnier	invent	
sunniest	invents	
teach	invented	
taught	inventing	
teacher	invention	
teachers	inventions	
teaches	invite	
teaching	inviting	
thai	invites	
thais	invited	
thailand	invitation	
thank	invitations	
thanks	laugh	
thanking	laughs	
thanked	laughing	
three	laughed	
third	laughter	
thirteen	loud	
thirteenth	loudly	
thirty	loudest	
thirtieth	louder	
two	magic	
second	magician	
twelve	magicians	
twelfth	malaysia	
twenty	malaysian	
twentieth	malaysians	
use	mean	
used	means	
useful	meant	
uses	meaning	
using	meaningless	
village	meanings	

villager	open	
villagers	opening	
villages	openings	
visit	opens	
visited	opened	
visiting	own	
visitor	owner	
visitors	owners	
visits	owns	
	owning	
	owned	
	peace	
	peaceful	
	pollute	
	pollutes	
	polluted	
	polluting	
	pollution	
	prepare	
	preparation	
	preparations	
	prepared	
	prepares	
	preparing	
	press	
	pressed	
	presses	
	pressing	
	pressure	
	pressures	
	protect	
	protected	
	protecting	
	protection	
	protections	
	protects	

	relation<br>relations<br>relationship<br>relationships<br>report<br>reported<br>reporter<br>reporters<br>reporting<br>reports<br>serious<br>seriously<br>serve<br>servant<br>servants<br>served<br>serves<br>serving<br>service<br>services<br>simple<br>simpler<br>simplest<br>simply<br>succeed<br>succeeded<br>succeeding<br>succeeds<br>success<br>successes<br>successful<br>talent<br>talented<br>talents<br>tour<br>tours	

	touring<br>toured<br>tourist<br>tourists<br>travel<br>traveled<br>traveling<br>travelled<br>traveller<br>travellers<br>travelling<br>travels<br>traveler<br>travelers<br>understand<br>understanding<br>understands<br>understood<br>win<br>winner<br>winners<br>winning<br>wins<br>won<br>wonder<br>wondered<br>wonderful<br>wondering<br>wonders<br>stomach<br>stomachs<br>stomachache<br>stomachaches	

Table 8: Proper Nouns

Grade 7	Grade 8	Grade 9

Dragon Boat Festival	Central Park	Water Festival
Alice	Huangguoshu Waterfall	Mid-Autumn Festival
Bob	Weld Quay	Mother’s Day
Cindy	Penang Hill	Father’s Day
Dale	Tian’anmen Square	A Christmas Carol
Eric	the Palace Museum	Santa Claus
Frank	American Teenager	Stonehenge
Grace	American Idol	World War II
Helen	America’s Got Talent	Men in Black
Gina	China’s Got Talent	Kung Fu Panda
Jenny	Mickey Mouse	Titanic
Brown	Steamboat Willie	March of the Penguins
Alan	Hollywood	Spider-Man
Tom	the Hollywood Walk of Fame	WildAid
Mike	The Old Man and the Sea	Annie
Jack	Animal Helpline	Alexander Graham Bell
Mary	World Trade Center	Clara
Miller	Journey to the West	Charles Dickens
Linda	the Monkey King	Scrooge
Jane	Sleeping Beauty	Jacob
Green	Cinderella	Tim
Smith	Little Red Riding Hood	Paula
Sally	Hansel and Gretel	Alfred
Kate	the Pacific Ocean	Billy
Paul	Qomolangma	Candy
Anna	the Nile	Jerry
John	the Caspian Sea	Emily
David	the Sahara	Marcus
Bill	the Yangtze River	Pam
Liza	the Yellow River	Roy
Jill	the Ming Dynasty	Whitcomb Judson
Peter	the Ming Great Wall	Ruby
Rick	the Himalayas	Thomas Watson
Jim	the Amazon	George Crum
Scott	Chengdu Research Base	James Naismith
Tony	The Beatles	Picasso
		Carla
		J.K.Rowling
		Victor
		Jean
		Paul Stoker
		Carmen
		Dan Dervish
		Maria
		Katie

Dave<br>Clark<br>Amy<br>Molly<br>Julie<br>Becky<br>Steve<br>Laura<br>Joe<br>Jeff<br>Johnny<br>Dean<br>Tina<br>Jackson<br>Carol<br>Lucy<br>New York<br>South Africa<br>the United States<br>Moscow<br>Toronto<br>Boston<br>the UK	Treasure Island<br>Alice in Wonderland<br>Little Women<br>Oliver Twist<br>Robinson Crusoe<br>Tom Sawyer<br>Harry Potter<br>Nashville<br>Country Music Hall of Fame Museum<br>National Science Museum<br>International Museum of Toilets<br>Hangzhou National Tea Museum<br>Donald Duck<br>Disneyland<br>Disney Cruise<br>the Terracotta Army<br>the Bird's Nest<br>Night Safari<br>Mark<br>Claire<br>Sue<br>Tara<br>Sam<br>Nelly<br>Larry<br>Greg<br>Danny<br>Eliza<br>Vera<br>Dennis<br>Sarah<br>Walt Disney<br>Minnie	Sato<br>Marie<br>Teresa Lopez<br>Marc LeBlanc<br>Bert<br>Holly<br>Matt<br>Kevin<br>Carl<br>Orson<br>Jason<br>Hayes<br>Jessica<br>Luke<br>Brian<br>Griffin<br>Trent<br>Macao<br>Chiang Mai<br>San Francisco<br>Berlin<br>Cali<br>Lausanne<br>New Zealand

	Andy<br>Ken<br>Hemingway<br>Kelly<br>Nick<br>James<br>White<br>Ted<br>May<br>Vince<br>Steen<br>Jake<br>Susan<br>Karen<br>Ben<br>Michael<br>Laura<br>Mills<br>Robert<br>Hunt<br>Judy<br>Nancy<br>Mandy<br>Aron Ralston<br>Mario<br>Jimmy<br>Sandy<br>Cathy<br>Taylor<br>Allen<br>Martin Luther King<br>Claudia<br>Tenzing Norgay<br>Edmund Hillary<br>Junko Tabei<br>Alex	

	Garth Brooks<br>Hong Kong<br>Georgetown<br>Utah<br>Alabama<br>Tennessee<br>Singapore<br>Southeast Asia	

Table 9: Abbreviations Containing Special Symbols

Grade 7	Grade 8	Grade 9
P.E.<br>A.M.<br>P.M.		
